# Constant pH Molecular Dynamics Simulation of pH Effects on Amyloid‐β Structure, Dynamics, and Metal‐Binding

**DOI:** 10.1002/chem.202500547

**Published:** 2025-05-28

**Authors:** Thuraya Albrahadi, Christelle Hureau, James A. Platts

**Affiliations:** ^1^ School of Chemistry Cardiff University Park Place Cardiff CF10 3AT UK; ^2^ Laboratoire de Chimie de Coordination – CNRS UPR8241 Université de Toulouse Toulouse 31000 France

**Keywords:** amyloid‐β, constant pH, copper, molecular dynamics, secondary structure, zinc

## Abstract

We report the first molecular dynamics simulations to examine the effect of pH on the structure, dynamics, and metal‐binding ability of amyloid‐β, the peptide implicated in the onset of Alzheimer's disease. We show that in the pH range of 6 to 8 only histidine residues show variable protonation, that predicted pKa values are in agreement with experimental data, and that changes in pH affect the size, flexibility, and secondary structure of the peptide. The binding of Cu(II) or Zn(II) to the peptide induces a shift of 1 to 1.5 pKa units in unbound histidine residues, while metal binding modes associated with higher pH induce significant changes in peptide structure. We speculate on the significance of these findings on results showing pH dependence as well as on Cu(II) and Zn(II) modulation of aggregation of Amyloid‐β.

## Graphical abstract

Constant pH molecular dynamics simulations are used to examine the effect of pH on amyloid‐β and its complexes with Cu(II) and Zn(II). Changes in size and secondary structure are found, with suggestions for the origin of pH‐dependent aggregation.

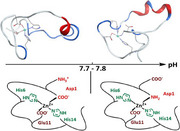

## 1 Introduction

Amyloid‐β (Aβ) is the principal peptide implicated in the onset of Alzheimer's disease (AD) and has been of interest to researchers for many decades. Naturally occurring mostly in 40 and 42 amino acid forms, it is an unstructured peptide that forms toxic oligomers and fibrils when it accumulates in the brains of AD sufferers. The reasons why Aβ changes from harmless monomer to toxic aggregate are not clear, and several hypotheses have been put forward to explain this key event, among which the amyloid cascade hypothesis is one of the most important.^[^ ^1^ ^]^

Two such hypotheses that are relevant to this work are i) that metal ions alter Aβ aggregation by changing the structure and dynamics of Aβ,^[^ ^2^ ^]^ and ii) that pH decrease in AD brains, potentially as a result of inflammation, acts to promote aggregation.^[^ ^3^ ^]^ Recently, Tian and Viles^[^ ^4^ ^]^ showed pH dependence of the primary nucleation phase of Aβ aggregation, with markedly faster assembly at lower pH values. In contrast, elongation and secondary nucleation were found not to be affected by pH (See Figure S1 for a reminder of key aggregation steps). This indicates that key effects of pH are likely to be found in monomeric and small oligomeric Aβ rather than in larger fibrils. While these experimental data are clear, there remains a question of how pH effects exert their influence on Aβ and its aggregation. Solubility will be vital, given that the pI of Aβ is 5.3 so greater aggregation is expected at pH values close to pI. However, one can also envisage changes in size, shape, flexibility, and secondary structure of Aβ as a function of pH that might affect aggregation.

Molecular dynamics (MD) is an ideal tool to examine such effects, but in standard MD methods, protonation states are chosen at the outset of a simulation and then fixed throughout. The popular AMBER family of forcefields offer different protonation states of histidine, including HIE and HID for higher pH and HIP for low pH, but these are probably too extreme to reflect the subtlety of effects at pH values close to the pKa of residues involved. A variant of traditional MD is the concept of “constant pH” MD, designed to allow the incorporation of pH effects into MD simulations.^[^ ^5^, ^6^ ^]^ In this approach, conventional MD is supplemented with regular (de)protonation steps evaluated using a Monte‐Carlo approach. This approach has been used successfully to predict specific pKa effects in proteins, but application to Aβ has not yet been reported. We have therefore employed constant pH MD to examine the effects of pH on structure, dynamics, metal binding, and aggregation of Aβ monomeric peptide.

**Scheme 1:**
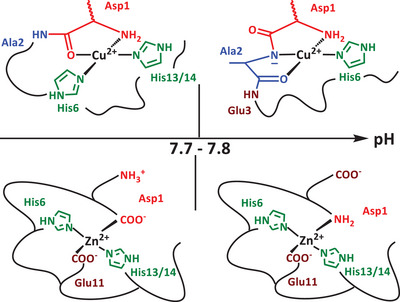
Variation in metal binding modes with pH. The apical position on the Cu(II) complexes is not shown for simplicity. Component I is the major species at lower pH values and component II at higher pH values. For simplicity, we do not distinguish between His binding via the N_δ_ or N_ε_ nitrogen atoms.

Moreover, another goal of this work is to evaluate whether constant pH MD can be used to predict the effects of metal‐binding on individual pKa values for specific residues within Aβ since it is possible that pH and metal effects may impact each other. Recently, Abelein and coworkers^[^ ^7^ ^]^ showed that at pH 8.0, the Cu(II) effects are mainly on the elongation rate of Aβ1‐42, acting to slow down elongation, thereby promoting the formation of amorphous aggregates at high metal:peptide ratio (>> 1) reminiscent of their previous proposal for Zn at highly sub‐stoichiometric ratios and pH 7.2.^[^ ^8^ ^]^ In addition, it is known that changes in pH result in altered metal binding modes for metal ions such as Cu(II) and Zn(II), with different coordinating residues found at pH below 7.7, and at pH above that value. The pH‐dependent Cu(II) and Zn(II) coordination sites to Aβ near neutral pH are reminded in Scheme 1, deduced according to seminal works referenced above; see also ref. [9] for an extended justification on the nature of the Cu(II) site at high pH, for which a controversy exists.^[^ ^10^ ^]^ Recent simulation work shows that incorporating Cu(II) and Zn(II) leads to major changes in peptide structure and flexibility,^[^ ^11^ ^]^ but to date has not addressed how these changes vary with high/low pH binding modes. Hence, we aim at delineating/identifying the possible changes in monomer structures upon metal binding but as a function of pH, to bring new insights into the role of Cu and Zn in Aβ self‐assembly modulation.

## 2 Methods

All simulations were performed using the AMBER22 package,^[^ ^12^ ^]^ using the tleap utility to construct parameters and CPU and GPU versions of pmemd for molecular dynamics. All peptides were initially in fully extended conformation to avoid biasing simulations. To facilitate a direct comparison between metals and binding modes, each metal ion in each component shown in Scheme 1 was bound to the extended peptide through the atoms shown, including the N_ε_ nitrogen atoms of each His residue involved. Metal ligand bonds were restrained to 2.0 Å to provide a starting point for detailed DFT determination of coordination geometry and forcefield parameters (vide infra).

Constant pH simulations used the ff10 forcefield^[^ ^13^ ^]^ with implicit generalised Born (igb = 2) solvation,^[^ ^14^ ^]^ as set out by Mongan et al. The use of implicit solvent means that no explicit water molecules enter the model. No cutoff of electrostatic interaction was used. Fixed protonation calculations compared standard Histidine protonation state (HIE) with doubly protonated form (HIP), with the same forcefield and solvent model for comparison. All constructed systems were first minimized for 1000 steps, then simulated with between 50 and 250 ns (and in one case, 1000 ns) production MD, typically in triplicate with different random starting velocity taken from Maxwell‐Boltzmann distribution at 310 K. All bonds to hydrogen were constrained, allowing 2 fs timestep for MD integration.

Analysis used the cpptraj program,^[^ ^15^ ^]^ part of AmberTools. Preliminary tests indicated essentially identical data from simulations using all titratable residues; all Asp/Glu were fully deprotonated and all Arg/Lys/Tyr were fully protonated at the pH values of interest, but with longer convergence times. We therefore proceed allowing only His residues to change protonation state. N‐terminus is positively charged throughout, while in full‐length peptide C‐terminus is negatively charged, whereas in truncated Aβ16 C‐terminus is capped with amide.

Simulation of metal‐Aβ complexes employed the metal center parameter builder^[^ ^16^ ^]^ (MCPB) tools to extract forcefield parameters from B3LYP/6‐31G(d)^[^ ^17^ ^]^ calculations in Gaussian09,^[^ ^18^ ^]^ which were integrated with ff10 parameters for peptides. Cu(II) and Zn(II) complexes were modeled as both component I, expected to dominate at pH values below ca. 8, and component II that dominates at higher pH values (*vide supra*). Constant pH calculations on metal complexes were performed only with component I forms of Cu(II) and Zn(II) bound to truncated Aβ16, since at the high pH values at which component II forms all His resides will be fully deprotonated. Cu component I complex was bound through the N‐terminal amine of Asp1, the carbonyl bond between Asp1 and Ala2, and the side chains of His6 and His14, while Zn component I was bound through sidechains of Asp1, His6, Glu11, and His14. At higher pH, Cu component II was bound through N‐terminal NH2, the deprotonated backbone between Asp1 and Ala2, the carbonyl between Ala 2 and Glu3, and the sidechain of His6. Zn component II was bound through N‐terminal NH2 of Asp1 and sidechains of His6, Glu11, and His14 (Scheme 1). Alternative coordinating residues (mainly with His13 replacing His14) yielded very similar results, as shown in Supporting Information. We note that this approach has been successfully used for many metal‐protein complexes, including several with Aβ.^[^ ^19^ ^]^

## 3 Results and Discussion

We start by examining pH effects in C‐terminally truncated Aβ1‐16 (DAEFRHDSGYEVHHQK‐CONH_2_), since this is known as the primary binding site for metal ions, as well as containing all three His residues in the sequence. pH changes were limited to these His residues since the pH range of interest in experiments (pH 6 to 8) does not allow protonation of Asp/Glu nor deprotonation of Lys/Arg/Tyr (data not shown).^[^ ^20^ ^]^ Constant pH simulations were performed in triplicate runs of 50 ns each, following minimization and 5 ns thermalization and equilibration. At each saved frame of those trajectories, the cphstats utility program was used to determine the protonation state of each titratable histidine residue, which was then summed into an average over the whole 150 ns of simulation. The same procedure was repeated for apo‐ form and for component I of Cu(II) and Zn(II) complexes. Table 1 reports the fraction of protonation of each Histidine residue calculated at a range of pH values, and the pKa estimated from the resulting trajectories (see Figure S2). pH values were selected to span the range from almost fully protonated to almost fully deprotonated in steps of 1.0, with one extra run at intermediate pH closest to estimated pKa that resulted from the first set of data (6.5 for free peptide, 5.5 for Cu & Zn). Standard deviations across triplicate simulations are generally small, indicating that sampling is sufficient for these relatively small peptides.

**Table 1:** Mean fraction of protonated His residues in Aβ1‐16, standard deviation in parentheses.

pH	apo‐His6	apo‐His13	apo‐His14	Cu His13^[^ ^a^ ^]^	Zn His13^[^ ^a^ ^]^
4	0.998 (0.000)	0.998 (0.001)	0.997 (0.000)	0.985 (0.008)	0.982 (0.012)
5	0.986 (0.006)	0.982 (0.009)	0.972 (0.004)	0.783 (0.098)	0.805 (0.132)
5.5	–	–	–	0.692 (0.121)	0.478 (0.087)
6	0.850 (0.029)	0.866 (0.048)	0.798 (0.021)	0.441 (0.088)	0.127 (0.067)
6.5	0.688 (0.100)	0.682 (0.027)	0.574 (0.014)	–	–
7	0.413 (0.068)	0.544 (0.079)	0.396 (0.016)	0.049 (0.025)	0.020 (0.009)
8	0.068 (0.011)	0.070 (0.022)	0.073 (0.024)	0.007 (0.006)	0.004 (0.002)
pKa	6.8	7.1	6.7	5.9	5.5

^[a]^ Metals are bound as component I through His6 and His14, which are therefore not titratable: data reported for sole free His13 only; essentially identical results were obtained when His13 is bound to metal and His14 is free, see Supporting Information.

For apo‐Aβ1‐16, pKa values are found to lie in the range 6.7 to 7.1. This is close to the direct values found either by potentiometric studies^[^ ^20^ ^]^ or NMR titration^[^ ^21^ ^]^ and are in line with pH‐dependent Aβ1‐40 & Aβ1‐42 aggregation recently reported.^[^ ^4^ ^]^ We observe slightly higher pKa for His13 compared to 6 and 14, but the change is small and the standard deviation across triplicate runs (0.05 to 0.1) for pH values close to the predicted pKa indicates this is not statistically significant. Although these values are expected, they represent valuable benchmarks of the simulation protocol, forcefield, solvent model, etc that support application to larger and more complex systems.

**Figure 1:**
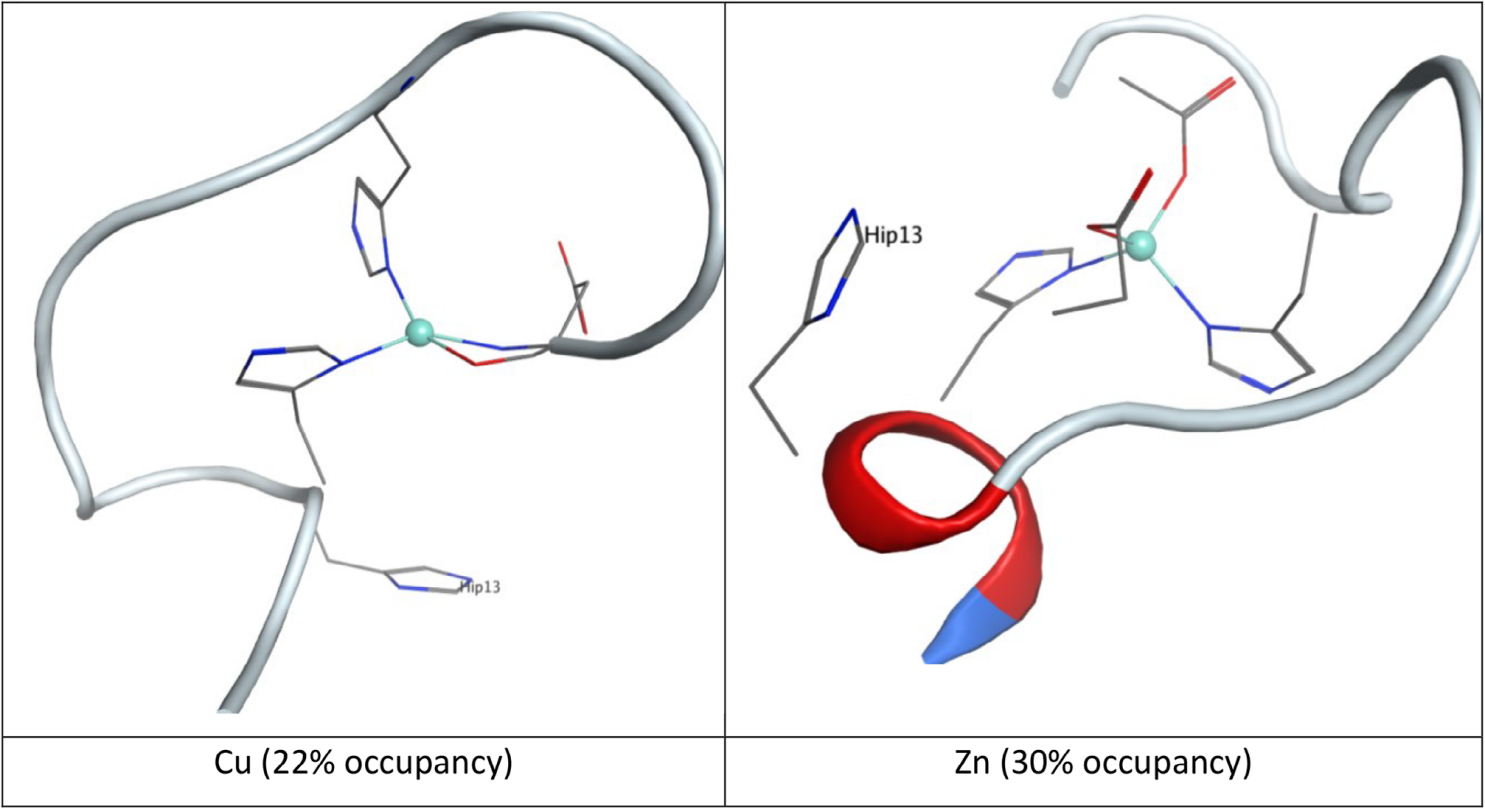
Most populated clusters of Cu‐ and Zn‐complexes of Aβ1‐16 at pH closest to pKa (6.0 for Cu, 5.5 for Zn). The metal ion is shown as a teal sphere, coordinating residues and His13 as lines, with hydrogens omitted for clarity. Histidine residues are shown as “Hip” since the constant pH MD method used requires this designation.

At the outset of this work, it was not clear whether constant pH methods would be compatible with the MCPB model required to include metal ions in simulations. To test this, we added Cu(II) and Zn(II) to Aβ1‐16 in component I form expected for lower pH, leaving a single His residue free in each case, for which protonation data is reported in Table 1. Firstly, it is important to note that the constant pH and MCPB approaches to MD simulation are fully compatible. Coordination modes to metal ions are identical to those reported previously for conventional MD, within the variation expected for stochastic simulations (Figure 1).

For Cu(II), predicted coordination geometry is also in good agreement with NMR values recently reported by Abelein et al.^[^ ^7^ ^]^ (Table S1) and more generally with the models proposed based on extensive spectroscopic studies and reported in refs. [10a–d] and reviewed in refs [2b–c]. Some minor differences exist, such as chirality induced by Asp1 binding and the nature of the binding nitrogen atom (N_δ_ or N_ε_) of the His bound to Cu(II). Here in the predominant form (Figure 1) Cu(II) is bound via the two N_δ_ of the His whereas it was reported that His6 binds through N_ε_.^[^ ^7^ ^]^ Of note, is the distortion from a perfect square planar, in line with data form Abelein; this allows for the possibility of a water molecule lying in between Cu and Asp1 carboxylate. For Zn(II), the model agrees well with that deduced from the recent available experimental data and summarised in Scheme 1.^[^ ^22^ ^]^ It differs from the Zn(II) site first proposed, where the three His were bound together with the deprotonated N‐terminal amine or the Asp1 carboxylate side‐chain.^[^ ^23^, ^24^, ^25^ ^]^

It is expected that both Cu(II) and Zn(II) induce significant changes in pKa of His13, the sole uncoordinated His residue in component I of both complexes. For the Cu complex, a shift of approximately 1 pKa unit is found, such that by pH6 this residue is predicted to be less than 50% protonated and by pH 7 just 5% protonated His13 is predicted. This is in line with the reported value obtained by potentiometry, which finds an average decrease of 1.1 to a pKa value for the free Histidine of 5.3.^[^ ^18^ ^]^ The effect of Zn(II) binding is even more pronounced, inducing a change of around 1.5 pKa units, making the sole free histidine in this Zn‐peptide complex strongly acidic with only around 13% protonation at pH 6 and 2% at pH 7.

That placement of a +2 cation in the vicinity of the free His residue reduces protonation is expected on electrostatic grounds, but quantitative predictions of this effect have not been previously reported. All‐atom simulation allows us to examine the distance between the metal ion and His13, which is approximately 9 Å in the extended starting geometry used. At the pH closest to pKa (6.0 for Cu, 5.5 for Zn) this distance falls to an average of 7.5 Å for Cu, and 6.0 Å for Zn (Figure S3). Figure 1 shows the most populated cluster from each set of trajectories, showing that His13 remains in the vicinity of the metal in both cases. In these clusters, the contact distance between the metal and free His is 7.1 Å for Cu, while for Zn the metal‐His distance is 6.6 Å. The closer distance for Zn suggests a possible reason for the larger shift in pKa for this ion compared to Cu, although both are clearly outer‐sphere coordination effects, since distances are never less than 5.5 and 4.5 Å for Cu and Zn, respectively, compared to direct coordination distances of ca. 2.0 Å.

Table 1 shows that His residues in Aβ are most likely to have variable protonation states between pH 6 to 8, so we carried out larger, longer constant pH simulations on the full‐length Aβ40 peptide at each of pH = 6, 7, and 8. Here, triplicate runs were heated to 300 K and equilibrated over 5 ns, then production simulations run for 220 ns with the first 20 ns omitted from reported data to account for further equilibration (see Figure S4 for RMSD to confirm pseudo‐equilibration in this time), resulting in 600 ns data for each pH value, 1.8 µs in total. These simulations result in similar protonation and pKa data (Table 2) to those found for the truncated peptide above. pKa values are again in the range 6.6–7.0, with less than 50% of residues protonated at pH 7 for all three His residues. This indicates that interactions between the N‐terminal fragment that contains His and the remainder of the 40‐residue peptide do not strongly affect pKa values in the full‐length monomeric peptide, and hence that the truncated peptide is a good model of the full‐length form. This agrees with the reported flexibility of the N‐terminal part of the peptide and the observation that salt‐bridge interactions are mainly gathered in the N‐terminal part of the peptide.^[^ ^26^ ^]^

**Table 2:** pKa of selected groups, fraction protonated, and overall charge for Aβ40.

	mean pKa [sd]^[^ ^a^ ^]^	Protonation pH6	Protonation pH7	Protonation pH8
His6	6.94 (0.52)	0.90	0.46	0.10
His13	6.73 (0.22)	0.86	0.26	0.08
His14	6.55 (0.39)	0.81	0.24	0.05
N‐term NH2	8.0^[^ ^b^ ^]^	0.99	0.90	0.5
Overall charge		−0.42	−1.91	−2.77

^a)^ Calculated over all 9 MD simulations;

^b)^ from ref [20].

**Figure 2:**
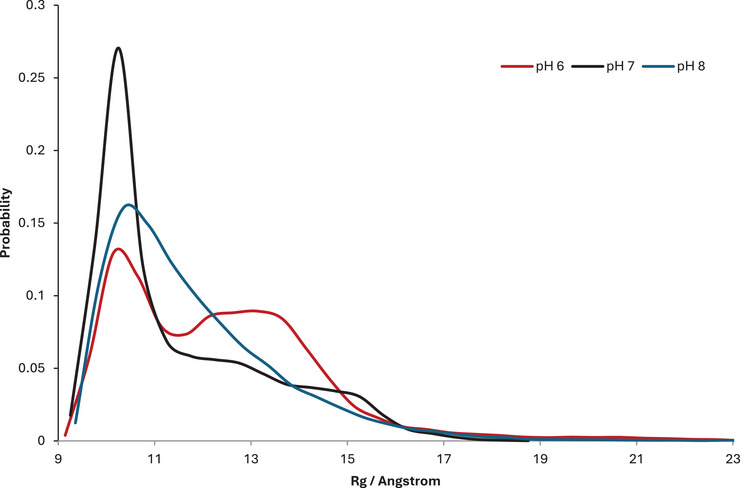
Histogram of radius of gyration (Rg) values of apo‐Aβ1‐40 at different pH values, obtained from 600 ns constant pH simulation.

Changes in pH lead to significant changes in the spatial extent of the peptide, as measured by the radius of gyration (Rg). Table 3 shows that at pH 6, Rg is on average slightly larger than at higher pH values, and is also more variable, with a larger standard deviation than at higher pH. Minimum Rg at each pH is very similar, but at low pH, the maximum value is notably larger, while at pH 7 this value is much smaller. These aspects are more evident in Figure 2, a histogram of Rg values across equilibrated simulations at each pH; all data exhibit the largest peak around 10 Å, but this is much narrower and more pronounced at pH 7, with small shoulders around 13 and 15 Å. In contrast, the shoulder peak around 13 Å is almost as prevalent as the smaller one for pH 6, while at pH 8 a broader, smoother spread of Rg values is evident with no apparent shoulder.

**Table 3:** The radius of gyration of Aβ1‐40 at different pH values (Å).

	pH6	pH7	pH8
Mean	12.62	11.81	12.08
sd	2.12	1.81	1.80
Min	9.14	9.25	9.36
Max	26.19	19.10	23.88

To exemplify the range of structures, snapshots that represent each of the regions of Rg, taken from the simulations at pH 6, are shown in Figure S5. The smallest structure exhibits a fold that brings the N‐ and C‐termini into close proximity (Rg = 10 Å) with the C‐terminal carboxylate, and small elements of helical and turn structures. An intermediate structure, with Rg = 13 Å, contains an anti‐parallel β‐strand formed between Leu17‐Val18 with Gly29‐Ala30, along with a short α‐helix in the N‐terminus (Ser8‐His14), but no apparent tertiary fold joining these elements of secondary structure. The largest structure has no defined secondary structure and adopts an almost fully extended form.

**Figure 3:**
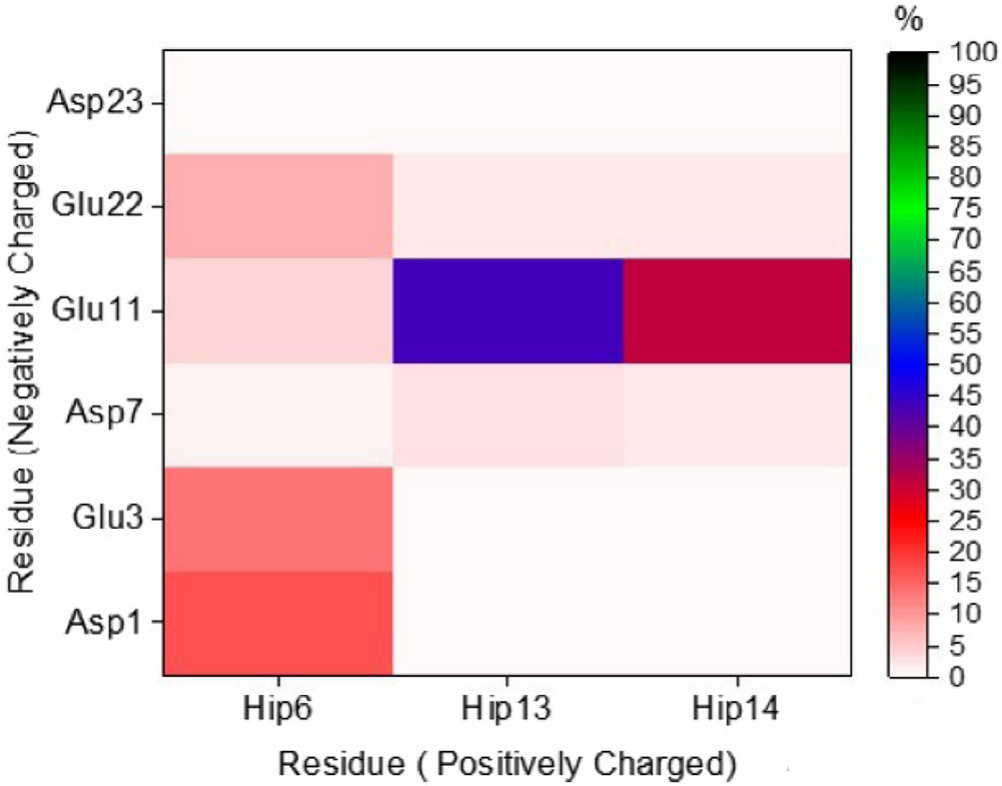
Percentage occurrence of salt‐bridge interactions at pH 6 between His and COO^−^ containing residues in apo‐Aβ1‐40 at pH 6, obtained from 600 ns constant pH simulation.

The importance of salt‐bridge interactions involving protonated His residues at pH 6 is shown in Figure 3, the percentage occurrence of these interactions across the whole 600 ns of equilibrated trajectories. We find that no such interactions are present for more than 50% of the time and that the most prevalent are relatively local, especially His13 with Glu11, but also His14‐Glu11 and His6‐Asp1. However, there are also some frames in which nominally distant residues form contacts, for example, His6‐Glu22, which is present for approximately 8% of frames. As expected, these His salt‐bridges are markedly less prevalent at pH7 and almost completely absent at pH8 (Figure S6).

**Figure 4:**
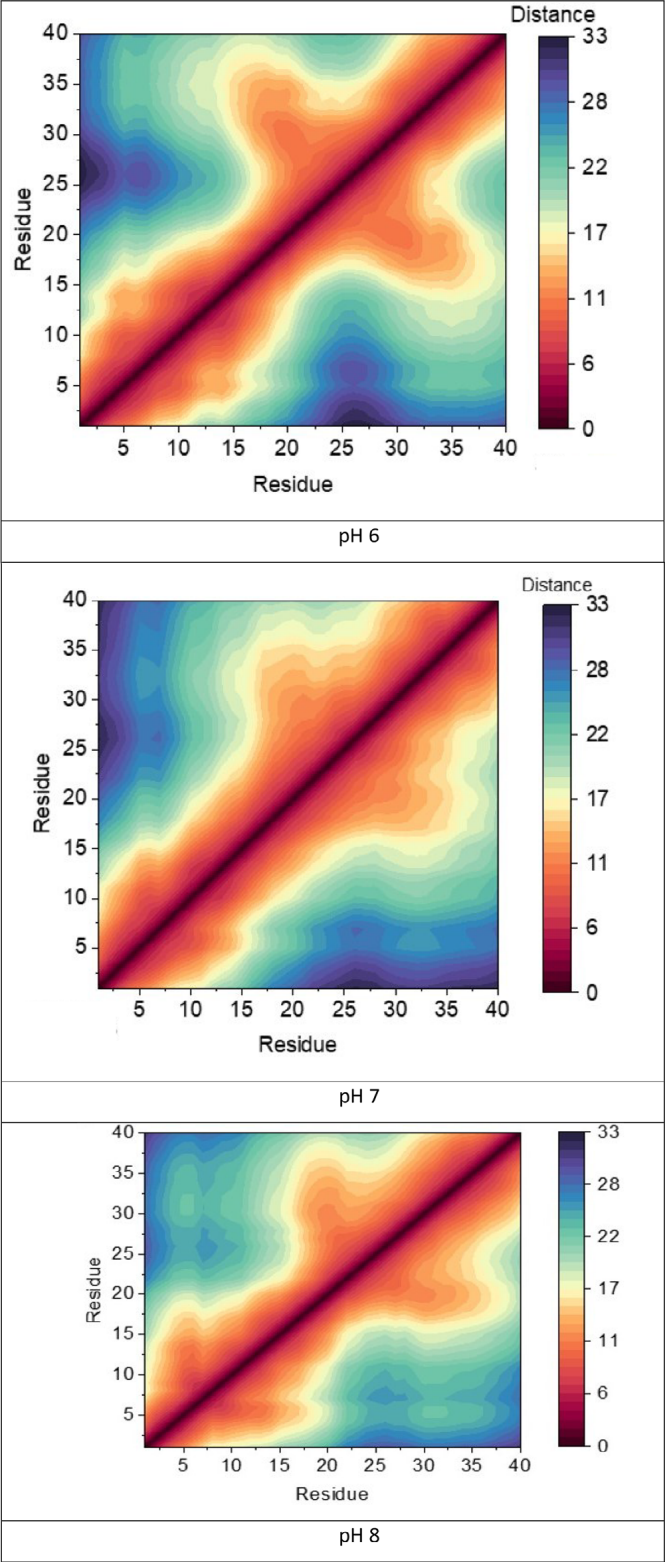
Inter‐residue contact maps of apo‐Aβ1‐40 at different pH values, obtained from 600 ns constant pH simulation (scale in Å).

As well as ionic interactions, more general inter‐residue contact plays a role in determining the conformations of Aβ. To examine this further, contact maps of the mean distance between Cα of each residue, averaged across 600 ns trajectories at each pH, are plotted in Figure 4. At pH6, we find evidence not only of persistent interactions in the N‐terminus but also relatively close contact in the C‐terminal region between Ala30‐Met35. We ascribe the former to enhanced salt‐bridges when His are protonated, and the latter to the fold noted in the compact structure in Figure S5. At pH7 there is significantly reduced inter‐residue contact in both N‐ and C‐termini, while at pH8 these interactions are again more evident.

The narrower spread of conformations at pH 7 is also reflected in root mean square fluctuation (RMSF), as shown in Figure S7, in which residues 7–18 and 30–36 are markedly less mobile at pH 7 than at either 6 or 8. Interestingly, the most marked differences in RMSF as a function of pH are seen in residues whose protonation does not change, such as Gly9 and Val12, and even in those quite remote from the N‐terminus such as Ile32‐Gly33.

**Figure 5:**
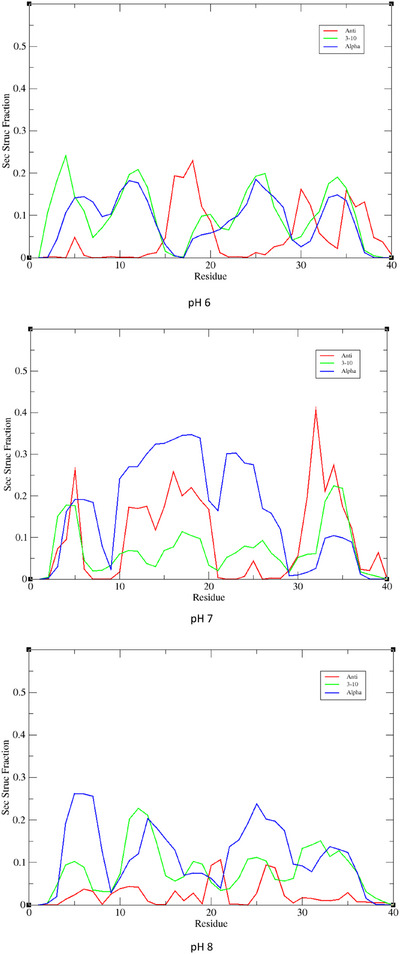
Secondary structure propensity of apo‐Aβ1‐40 at different pH values, expressed as a fraction of frames from 600 ns MD simulation.

The effect of pH on peptide structure is further evident in the secondary structure, as shown in Figure 5. At pH 6 we find a picture of a relatively unstructured peptide, with no form of secondary structure existing for more than *ca*. 20% of frames. Helical character, in the form of α‐ and 3,10‐helices, are evident in the N‐terminal region in particular, while anti‐parallel β‐strands are seen in the central hydrophobic core and C‐terminus (*cf* Figure S5). Increasing pH to 7 results in a greater proportion of α‐helices, now shifted towards the center of the peptide, while β‐strands are also more evident across the whole sequence. This trend is reversed at pH 8, which more closely resembles pH 6 than 7, but with only α‐helices present at greater than 10% of frames.

**Figure 6:**
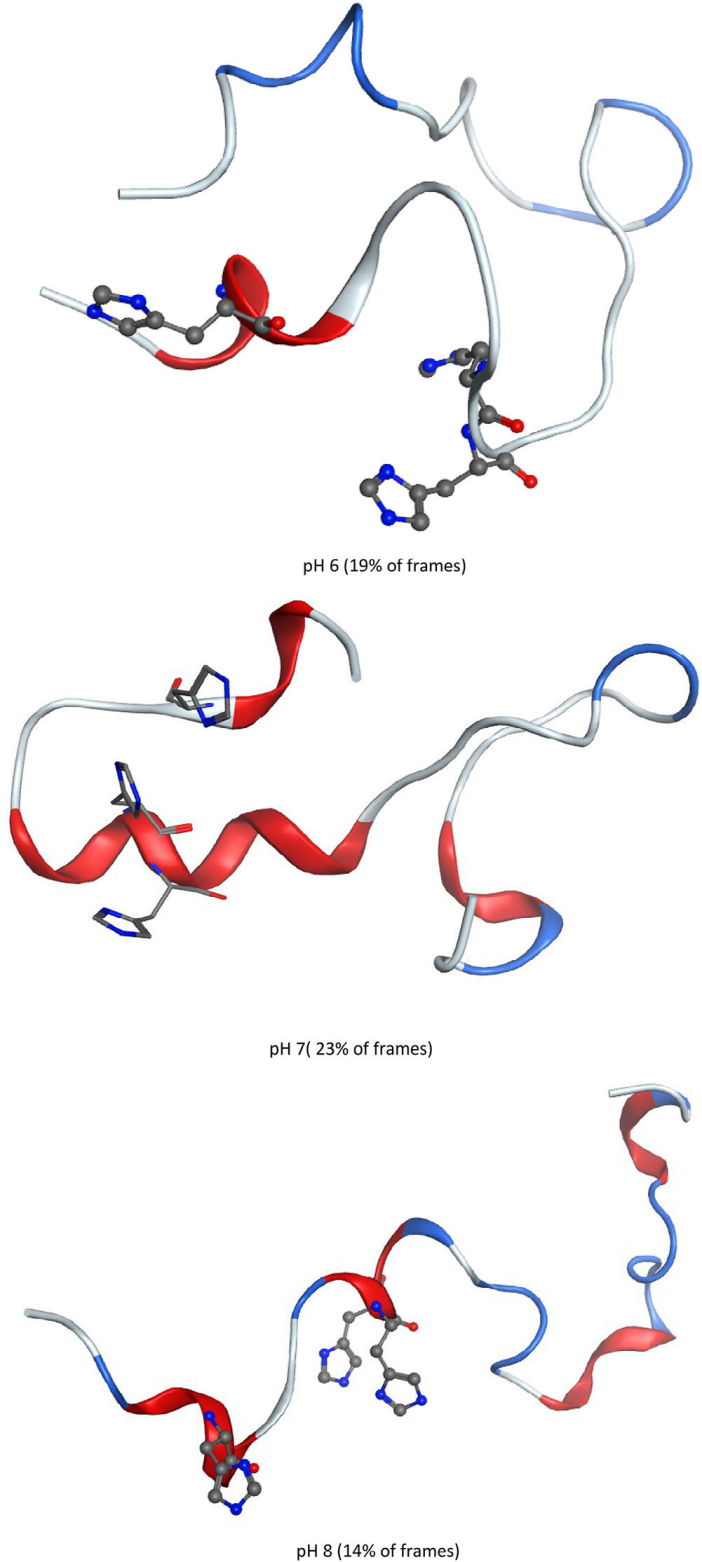
A most populated cluster of Aβ40 at different pH; red: helix, blue: turn, white: coil. Histidine residues are shown as ball‐and‐stick, without hydrogens for clarity: all His are contained in the N‐terminal region.

This picture of structural change resulting from pH differences is further shown by views of the most populated clusters across the whole trajectory at each pH (Figure 6). At pH 6, the largest cluster (representing 19% of frames) shows very little structure, with just a short, isolated helical‐type form close to His 6. At pH 7 (23% of frames), however, a much larger and more evident α‐helix is found encompassing His13 and His14, along with smaller ones at N‐ and C‐termini and turn‐like structures between these. The pH 8 cluster (14% of frames) is intermediate between these, with numerous short helical and turn structures evident across the whole peptide. No β‐strand structure is apparent in any of these most populated clusters, and the short, isolated nature of the helices found agrees with the accepted view of this as an unstructured peptide.

Similar views of the next nine clusters are shown in Supporting Information (Figure S8). For all three pH values, we find that the next most populated cluster, accounting for 14–15% of frames, exhibits significant β‐strand structure in the CHC and C‐terminus. Similar features are found in several other clusters, such that in total 38% of frames exhibit strand‐like structures at pH 6, 39% at pH 7, and 47% at pH 8.

The most populated cluster at pH 6 was also used to interrogate potential ionic interactions between protonated His residues and Asp/Glu in the chain. We find that His6 forms close contact with both Asp1 (2.71 Å) and the carboxylate C‐terminus of Val40 (3.28 Å), which seems likely to be the origin of the N‐ to C‐terminus contact noted above. No close contacts with anionic sidechains are found for His13, but His14 forms a close contact with Asp7 (2.74 Å). Views of these contacts are presented in Figure S9.

Our results show that the level of β‐strands in the Aβ1‐40 monomer, which remains similar within the studied pH range does not correlate with the higher propensity of Aβ1‐40 to form aggregates at lower pH values.^[^ ^4^ ^]^ This may indicate that folding of the monomer to a more aggregation‐prone structure is not the main determinant to explain the enhanced aggregation properties detected at lower pH values and that the formation of dimers and higher oligomers has to be considered as well.

The data in Table 1 indicate that it is unlikely that non‐coordinated His in metal‐Aβ complexes will be protonated to a significant extent at the pH values of 6 to 8. However, it is known that metal binding modes can vary with pH, so a further set of simulations was performed to compare Cu(II) and Zn(II) complexes with full‐length Aβ40 using both component I (lower pH) and component II (higher pH) coordination modes. In the light of the data in Table 1, the protonation state of non‐coordinated His13 was fixed as deprotonated. As above, given the greater size and flexibility of the peptide in this case, triplicate runs of 220 ns were used, with all other settings for MD simulation kept identical to the constant pH simulations. Numerical data was taken from 450 ns of data for each binding mode, discarding the first 70 ns of each run for equilibration since metal coordination places greater strain on the peptide structure.

**Figure 7:**
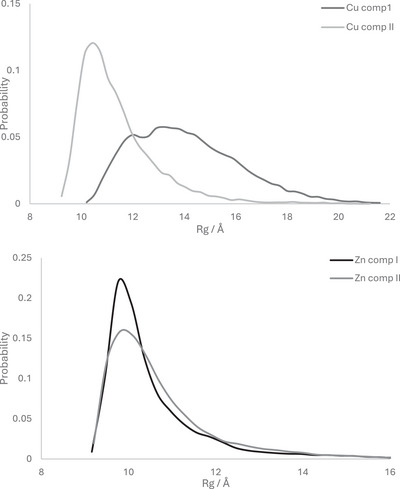
Histogram of radius of gyration (Rg) values of components I and II Cu and Zn complexes with Aβ1‐40, obtained from 600 ns constant pH simulation.

Table 4 summarises the size of each metal‐Aβ1‐40 complex, and indicates that most of the metal complexes of Aβ1‐40 exhibit similar mean Rg values to that observed at pH 7 for the metal‐free peptide. However, the component I Cu complex is markedly larger than the rest and also exhibits a larger standard deviation. This is also evident in histograms of Rg across the whole trajectory for each complex (Figure 7): three complexes exhibit a single main peak around 10 Å, with perhaps evidence for a shoulder in Cu component II at 11.5 Å. In contrast, Cu component I is much more extended, with peaks around 12 and 13.5 Å and a significant population out to 18 Å or more. To check this was not due to sampling error, one simulation was extended to 1 µs without significant change in mean Rg, either as an overall average or as a running average over 100 ns windows (see Figure S9).

**Table 4:** The radius of gyration of component I and II Cu and Zn complexes with Aβ1‐40 (Å).

	Cu component I	Cu component II	Zn component I	Zn component II
Mean	14.25	11.53	10.73	10.89
sd	2.09	1.52	1.24	1.32
Min	10.21	9.23	9.16	9.20
Max	24.34	21.37	19.45	20.55

**Figure 8:**
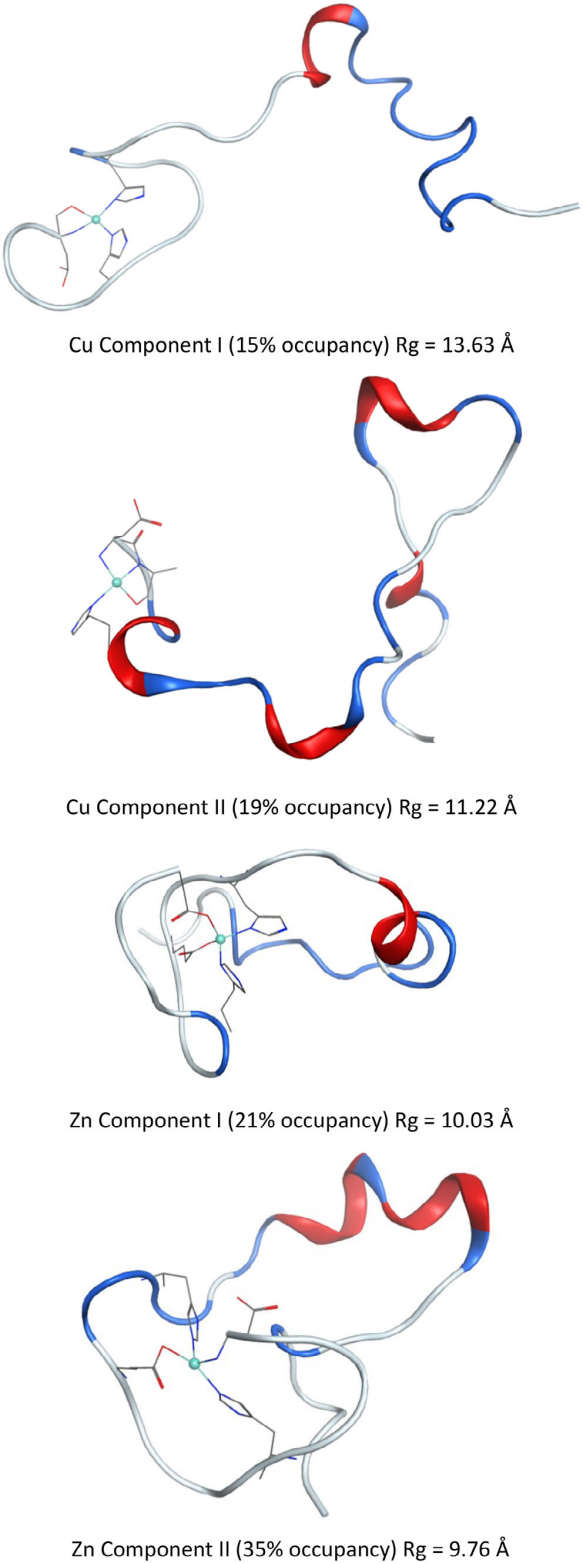
Most populated cluster of each type of metal‐Aβ1‐40 complex. Metal coordinating residues are shown, without hydrogens for clarity: all are contained in the N‐terminal region.

The greater spatial extent of Cu component I complex is also apparent in the most populated cluster across the trajectory (Figure 8), in which no defined secondary structure is apparent in the first N‐terminal residues, and mainly turn/bend is seen in the remainder (Rg of this structure = 13.6 Å). Similar features are evident in the remaining clusters (Figure S10), with the N‐terminal and central hydrophobic regions extended and unstructured and a mix of short helix and turn/bend in the C‐terminus. Only one cluster, representing 7% of the trajectory, contains a β‐strand structure. Cu component II and both Zn complexes contain more elements of secondary structure, including turn/bend in the metal binding region and longer helical sections, and folded tertiary structures, leading to notably lower Rg across the MD ensemble. No contacts between N‐ and C‐termini are found in either Cu(II) complex, but in both Zn(II) complexes we find backbone hydrogen bonds: in component I between Glu11 and Leu34, and in component II between Glu3 and Gly33.

**Figure 9:**
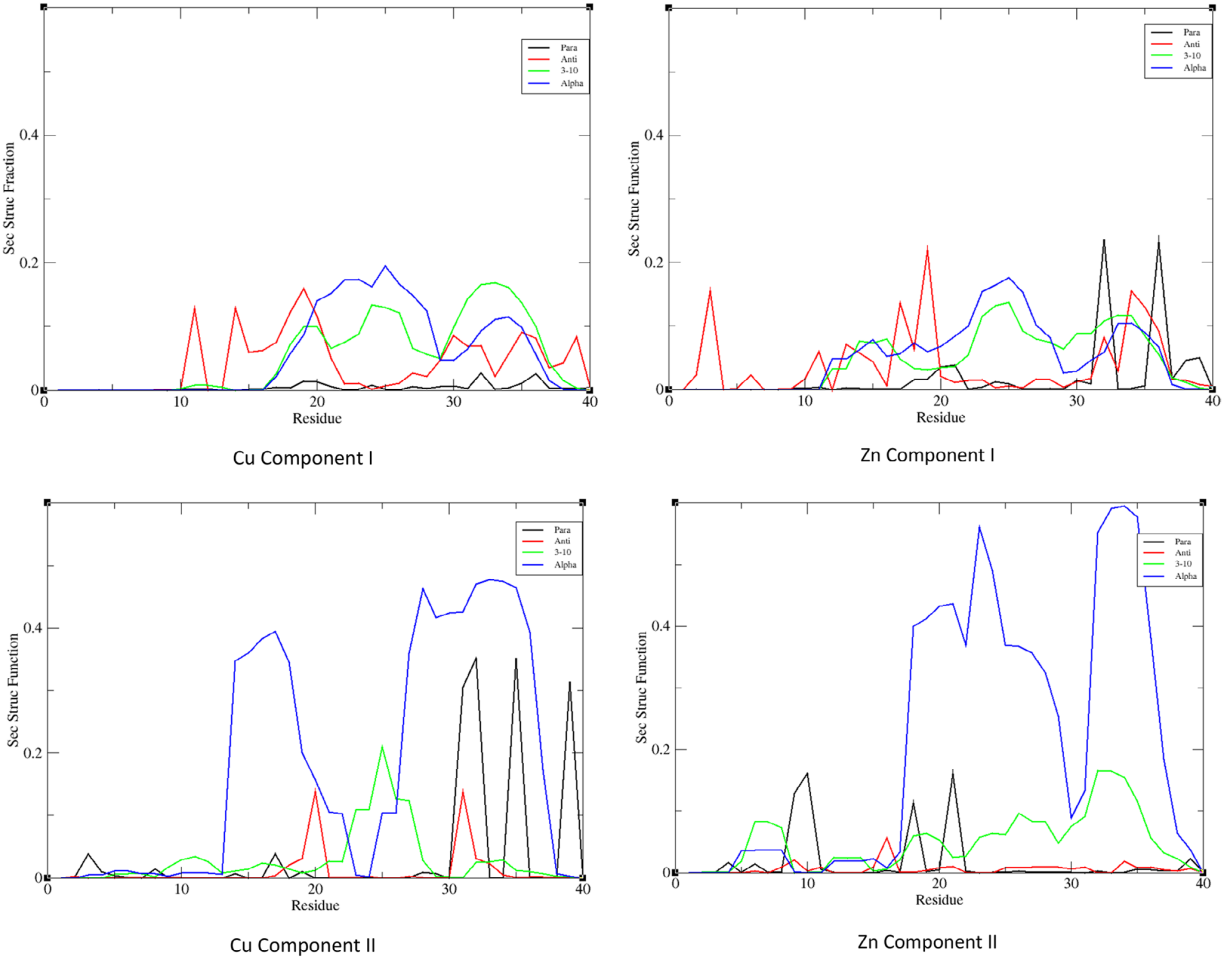
Secondary structure propensity of Cu and Zn complexes with Aβ40 expressed as a fraction of frames from 600 ns MD simulation.

The lack of structure in Cu component I is apparent in secondary structure propensity across the entire trajectory, as shown in Figure 9, where a helical percentage is never greater than 25% and a small element of anti‐parallel β‐turn is found in the N‐terminus. The higher pH component II structure, on the other hand, has substantial amounts of helical structure in CHC and C‐terminus, rising to 50% of frames, along with elements of β‐strands in C‐terminus. Zn component I also has no element of secondary structure over 25%, but displays sizeable amounts of both parallel and antiparallel β‐strands across the whole length of the peptide. Zn component II, like its Cu counterpart, has up to 50% of the trajectory in helical form in the C‐terminus, along with smaller amounts of β‐strand. Our results show that the level of β‐strands, higher for Zn(II) versus Cu(II) is in line with the reported data on metal‐modified Aβ1‐40 aggregation at neutral pH, in which Zn(II) has been shown to enhanced aggregation and Cu(II) to favor the formation of less defined assemblies. Our results show that the level of β‐strands, higher for Zn(II) versus Cu(II), is in line with the reported data on Cu(II) and Zn(II) modified Aβ1‐40 aggregation at neutral pH, in which Zn(II) has been shown to enhanced aggregation and Cu(II) to favor the formation of less defined assemblies.

The overall secondary structure content of each system studied, grouped into a helix (including α, π and 3,10 forms), β‐sheet (parallel and anti‐parallel), and other (turn, bend, and coil) is summarised in Table 5. In all simulations, the β‐sheet content is low, typically around 5% but rising to over 10% at pH7in the apo‐peptide. Helical content is higher at around 20% for most simulations, but falls to 10% in both component I forms of metal complex.

**Table 5:** Summary of secondary structure (%) and overall charge (e) of Aβ40.

		Sheet	Helix	Other	Charge
apo	pH6	6.4	18.6	75.0	−0.44
apo	pH7	10.4	23.1	66.5	−1.52
apo	pH8	4.4	19.8	75.8	−2.77
Cu	Comp I	4.8	10.9	84.4	−2.0 (pH 7)
Cu	Comp II	4.7	20.9	74.4	−3.0 (pH 8)
Zn	Comp I	5.8	10.1	84.1	−1.0 (pH 7)
Zn	Comp II	2.3	25.6	72.1	−2.0 (pH 8)

Also reported in Table 5 is the charge on each system: for the apo‐peptide this is as predicted from constant pH MD and so fractional, whereas for metal complexes protonation state was fixed throughout, and charge reflects the balance of metal ion and (de)protonated peptide backbone and/or side chains. As expected from the pI value of 5.3, the apo‐peptide is negatively charged at all pH values considered, and at pH 6 it is closest to zero while at pH 8 it is almost fully protonated with an overall charge close to −3.0.

## 4 Concluding Remarks

### 4.1 pH‐Dependent Aβ Self‐Assembly

We have shown that constant pH molecular dynamics simulations can shed light on the pH‐dependent aggregation behavior of Aβ, the peptide closely implicated in Alzheimer's disease. Predicted pKa values for the three histidine residues in the N‐terminal region are in close agreement with experimental data at between 6.6 and 7.0. Taken together, the results reported indicate that pH has a significant effect on the structure and dynamics of Aβ. At low pH, the monomeric peptide is relatively flexible and unstructured, a picture that changes to more structured and restricted at pH 7. One possible origin of this effect seems to be ionic interactions formed between protonated histidines and anionic residues in the N‐terminal region of the peptide, and the lack of such interactions with the central and C‐terminal regions. This might act to “wrap‐up” the aromatic histidines, preventing them from interacting with the rest of the peptide, leading to a more extended and less structured ensemble of conformations. However, we find no correlation between the structures found at low pH and secondary structure, particularly the amount of β‐sheet formed, and the known aggregation propensity at pH 6. Besides, it was recently proposed that the faster Aβ aggregation at lower pH can be attributed to a faster nucleation step rather than increased secondary and elongation rates.^[^ ^4^ ^]^ Taking that into account, our results suggest that the origin of differences in aggregation does not lie solely within the conformational ensembles adopted by the monomeric peptide. We thus proposed that changes in intermolecular interactions that may be facilitated by the decrease of the net charge when decreasing the pH (Tables 1 & 5) play an important role. We are currently investigating this possibility and hope to report a simulation of pH dependence of dimerization in due course.

### 4.2 Metal‐Modulation of His Protonation State

The introduction of a metal ion lowers the pKa of His protonation by between 1.0 and 1.5 pH units for Cu(II) and Zn(II), respectively for the non‐coordinated histidine. This suggests that these are likely to be fully deprotonated at pH 7, and that significantly more acidic conditions are needed to induce protonation of free His in complexes with metal(II) ions. Such a metal‐dependent effect was not fully expected according to the HSAB theory. Several reasons may explain the observed trend. First, apart from the intrinsic Lewis acidity of both ions, the nature of the coordination sphere matters and, in the present case, it is significantly different (Scheme 1) and this effect may dominate. Moreover, the apparent charge of the peptide bound to Cu(II) is lower by one unit versus that of the Zn(Aβ) complex due to the Cu(II) but not Zn(II)‐induced deprotonation of the N‐terminal amine (Table 5). In addition, the Cu(II) site remains relatively localized in the N‐terminal part of the peptide, apart from the rest of the peptide whereas the Zn(II) site is found buried in the whole peptide sequence (Figure 8). This could act to minimize the possibility of stabilizing interactions between His residues and the remainder of the peptide.

### 4.3 Metal‐Modulation of Aβ Self‐Assembly

For all complexes except Zn(II)(Aβ) component I, the N‐terminal amine was deprotonated and bound the metal center, in line with proposed models from spectroscopic experiments in Scheme 1. Beyond the involvement of the N‐terminal amine, the structures found here agree well with those deduced from spectroscopic data, as described in the main text. The simulations on the full‐length peptide complexes in low‐ and high‐pH forms show differences in size and flexibility: the component I form of the Cu(II) complex is particularly flexible and extended, whereas higher pH component II form of Cu(II) complex and both Zn(II) complexes are more compact and relatively rigid. In addition, the level of β‐sheet content does not strongly depend on the nature of the metal ion at play. This may contrast with the significantly distinct effects of Cu(II) and Zn(II) on Aβ‐assembly reported at neutral pH.^[^ ^2^, ^9^ ^]^ In addition, the low level of β‐sheet found might explain the discrepancies in the reported metal‐modulation of assembly data, in line with high sensitivity to external conditions such as type of buffer, ionic strength, etc. With respect to the apo‐peptide, the level of β‐sheet is weaker in the presence of metal ions regardless of the metal and component. This is in line with the Cu(II) or Zn(II)‐induced retardation of Aβ self‐assembly attributed to a slow‐down in the elongation step,^[^ ^7^, ^8^ ^]^ due to the formation of a conformation less prone to add at the end of the fibril. With respect to pH‐dependent effects on Cu(II) and Zn(II) modulation, only speculations could be drawn since no experimental data have been reported on that point. We are currently working on obtaining such aggregation data as well as on the modeling of the dimerization process in the presence of metal ions.

## Conflict of Interests

The authors declare no conflict of interest.

## Supporting information



Supporting Information

## Data Availability

The data that support the findings of this study are available from the corresponding author upon reasonable request.
