# Supplementary material for: Constant pH Molecular Dynamics Simulation of pH Effects on Amyloid‐β Structure, Dynamics, and Metal‐Binding
Source: Chemistry. 2025 May 28;31(34):e202500547. doi: 10.1002/chem.202500547 (PMC12172581; doi:10.1002/chem.202500547)

Supporting Information for

**Constant pH Molecular Dynamics Simulation of pH Effects on** **Amyloid-β Structure, Dynamics and Metal-Binding**

Albrahadi, Hureau and Platts

**Figure S1** Summary of key aggregation steps


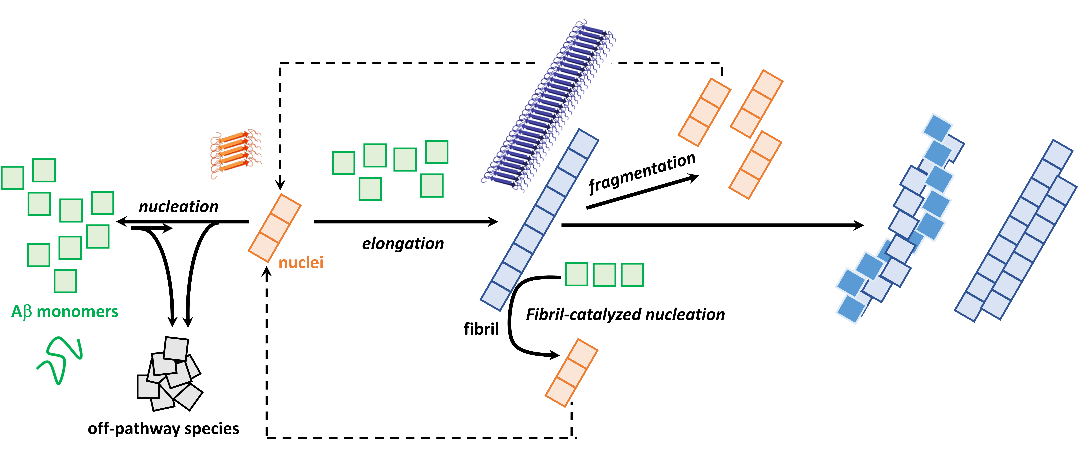


**Figure S2** Plots of fraction protonated as a function of pH from constant pH simulation of Ab1-16

| 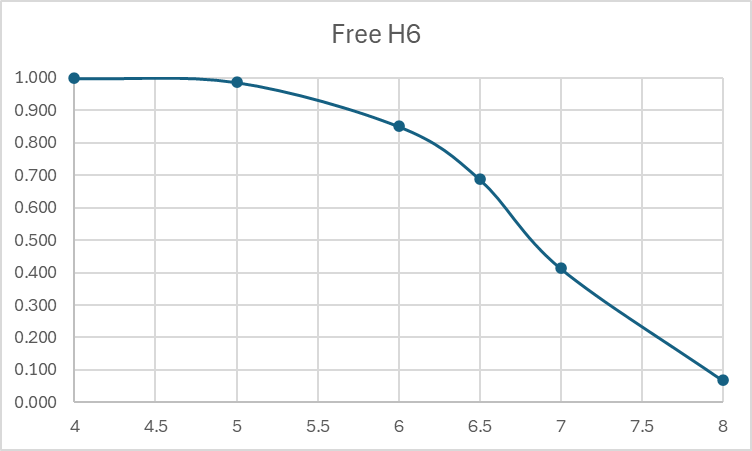 |
| --- |
| 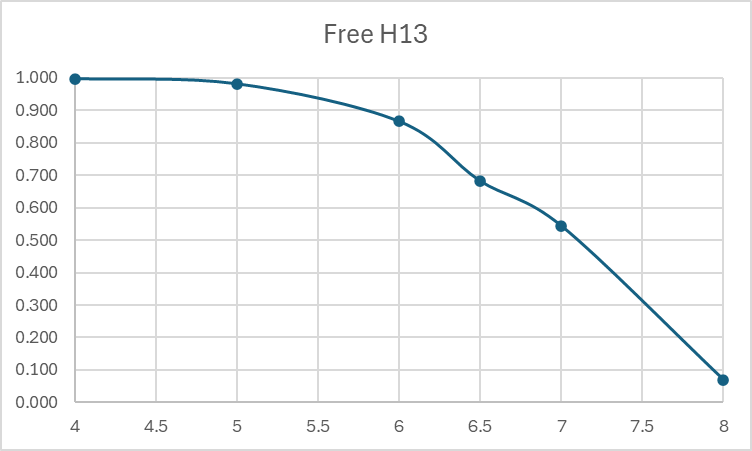 |
| 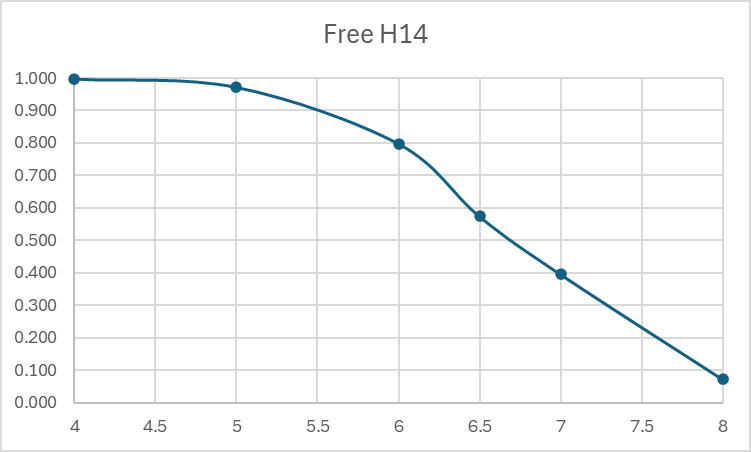 |
| 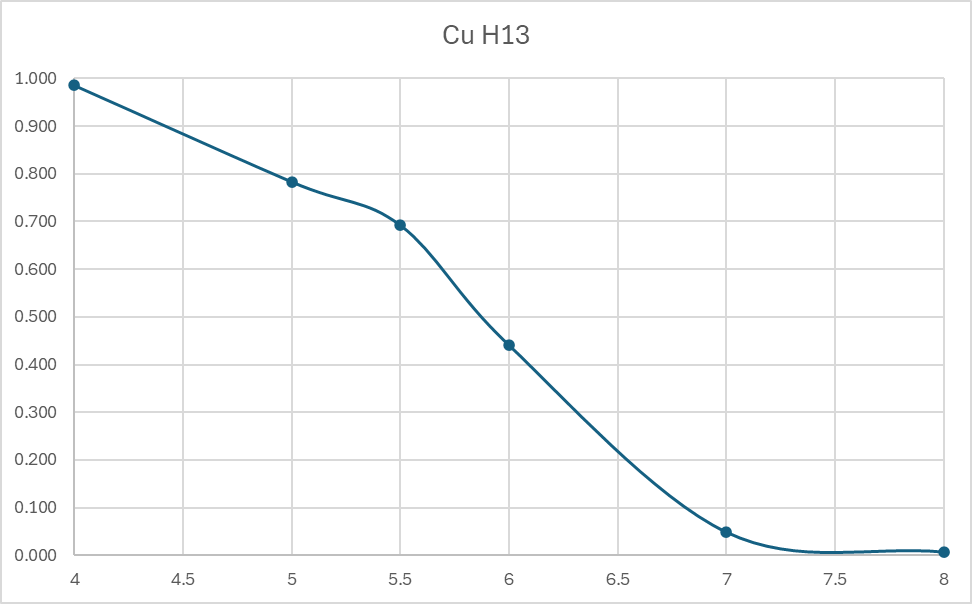 |
| 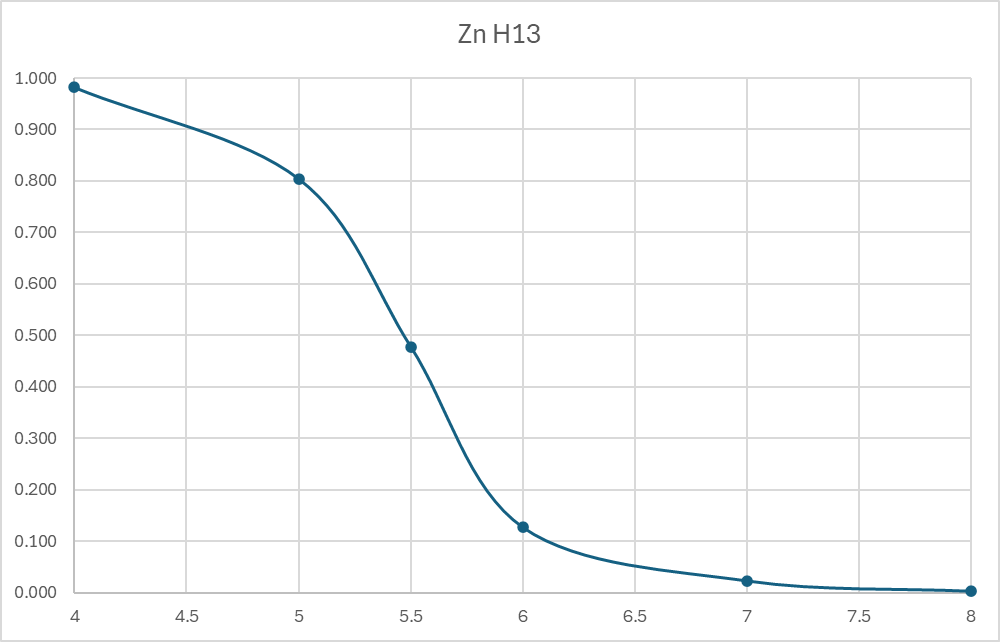 |

**Table S1** Comparison of NMR and MD metal-ligand distance

|  | NMR | MD |
| --- | --- | --- |
| Cu-N(Asp1) | 1.96, 1.96, 1.91, 1.94, 1.94 | 2.04 |
| Cu-O(Asp1) | 2.06, 2.19, 2.11, 2.15, 2.08 | 2.15 |
| Cu-N(His6) | 2.00, 2.03, 2.03, 2.02, 1.99 | 2.07 |
| Cu-N(His14) | 2.10, 2.16, 2.04, 2.13, 2.05 | 1.96 |

**Figure S3a** Metal-His13 distance at pH 6 for Cu-Aβ16 over 3 x 50 ns constant pH MD simulation


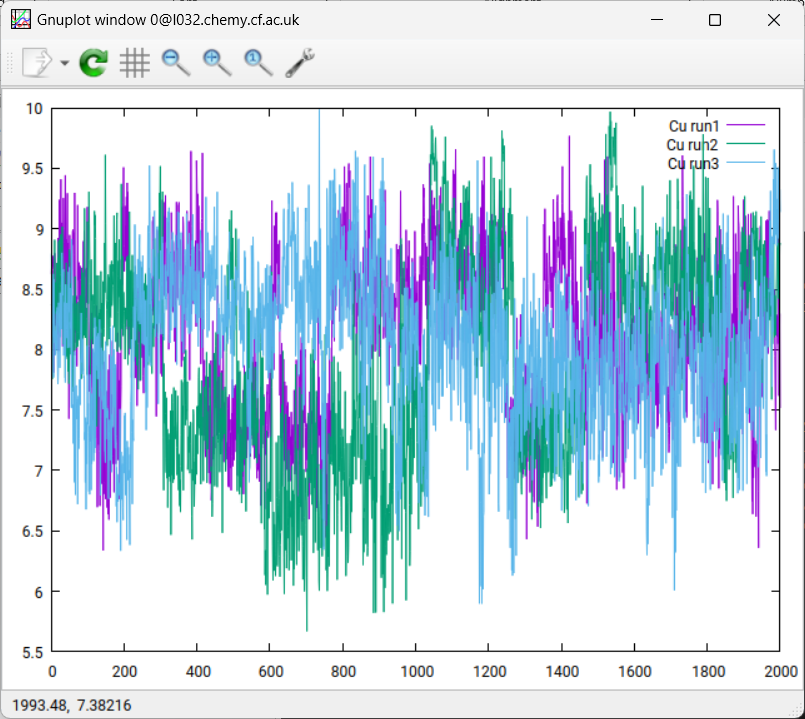


Metal-His13 / Angstrom

Frame

**Figure S3b** Metal-His13 distance at pH 5.5 for Zn-Aβ16 over 3 x 50 ns constant pH MD simulation


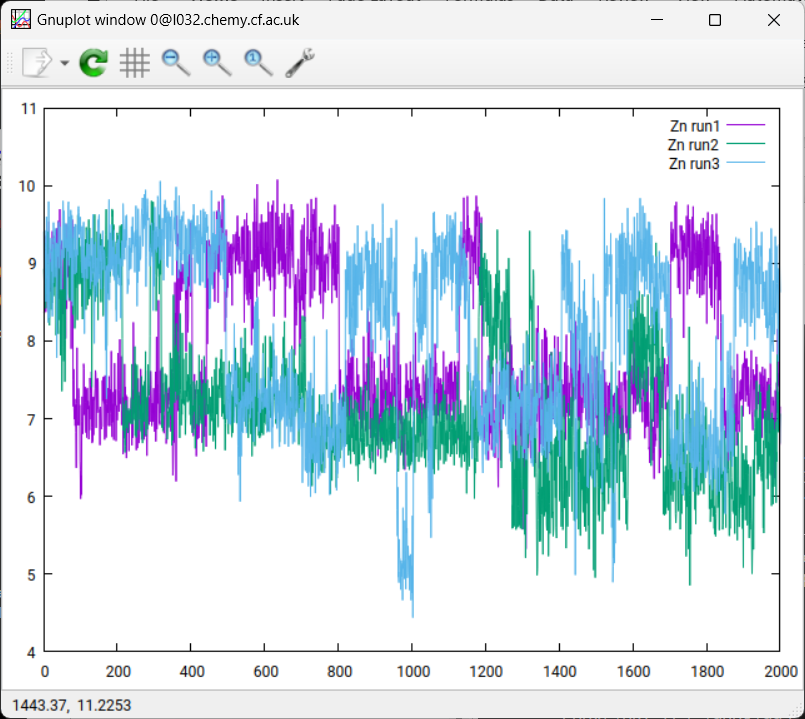


Frame

Metal-His13 / Angstrom

**Figure S4** RMSD for Ab40 at pH = 6, 7, 8

RMSD relative to minimised structure for 3 runs at pH6


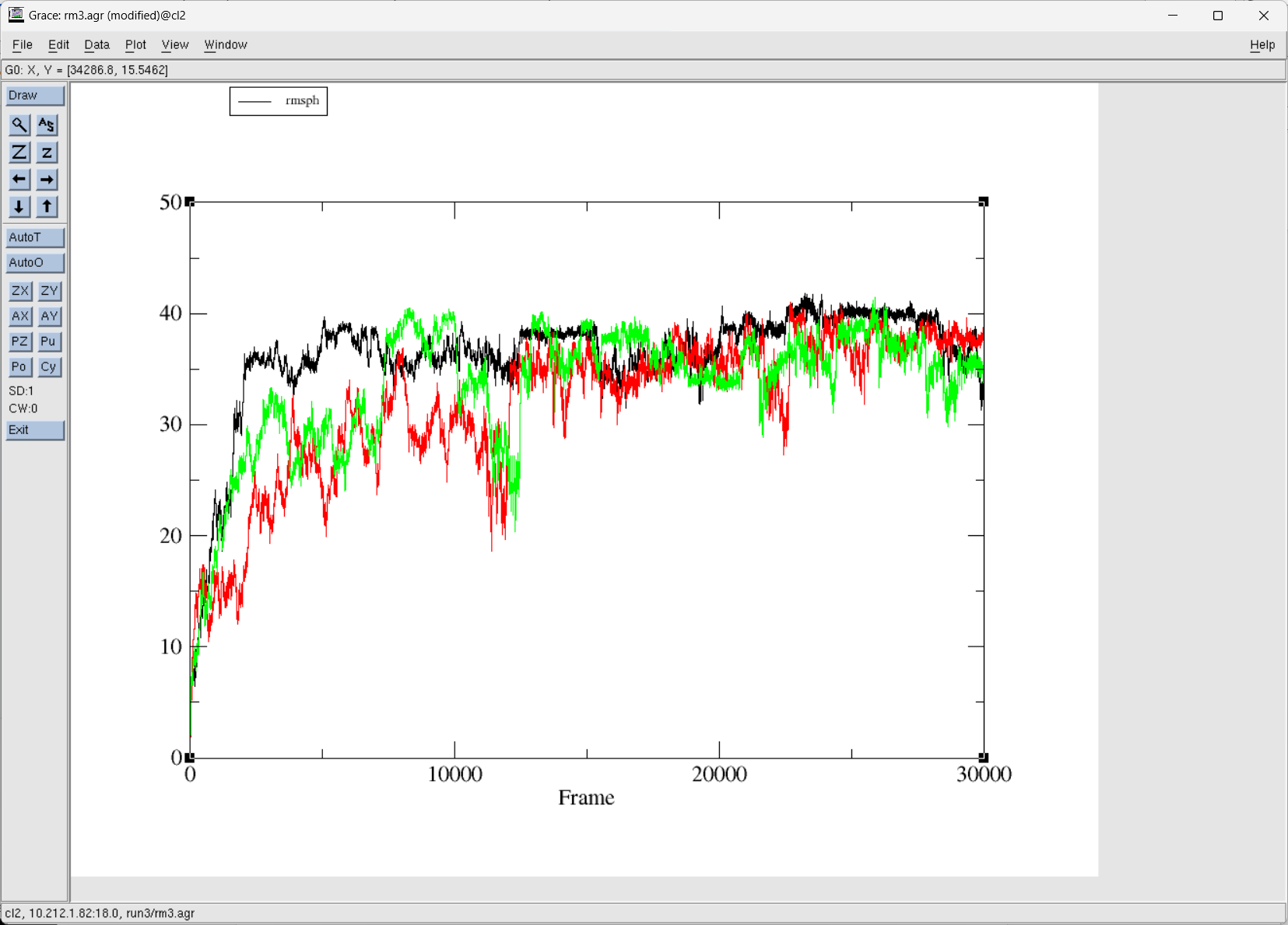


RMSD / Angstrom


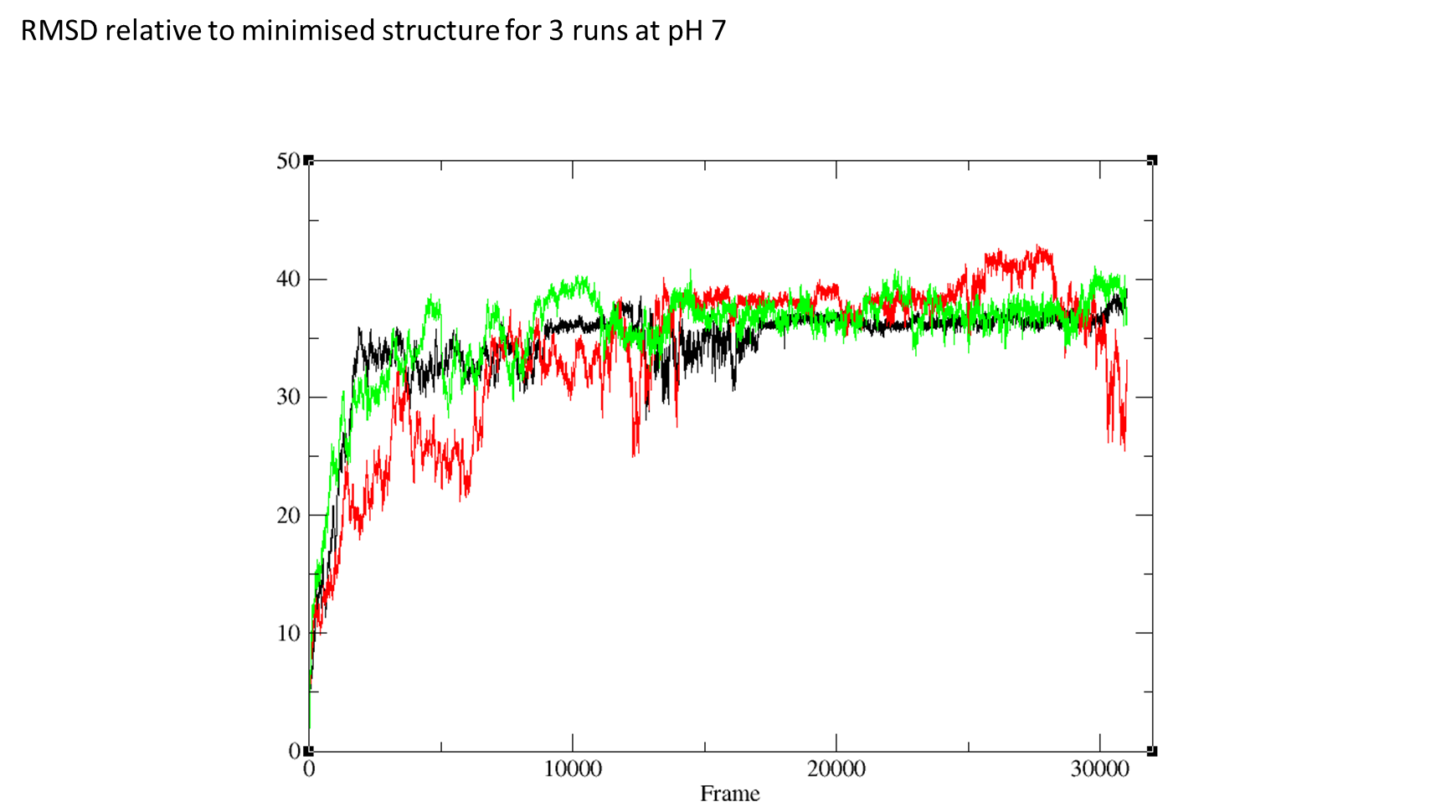


RMSD / Angstrom


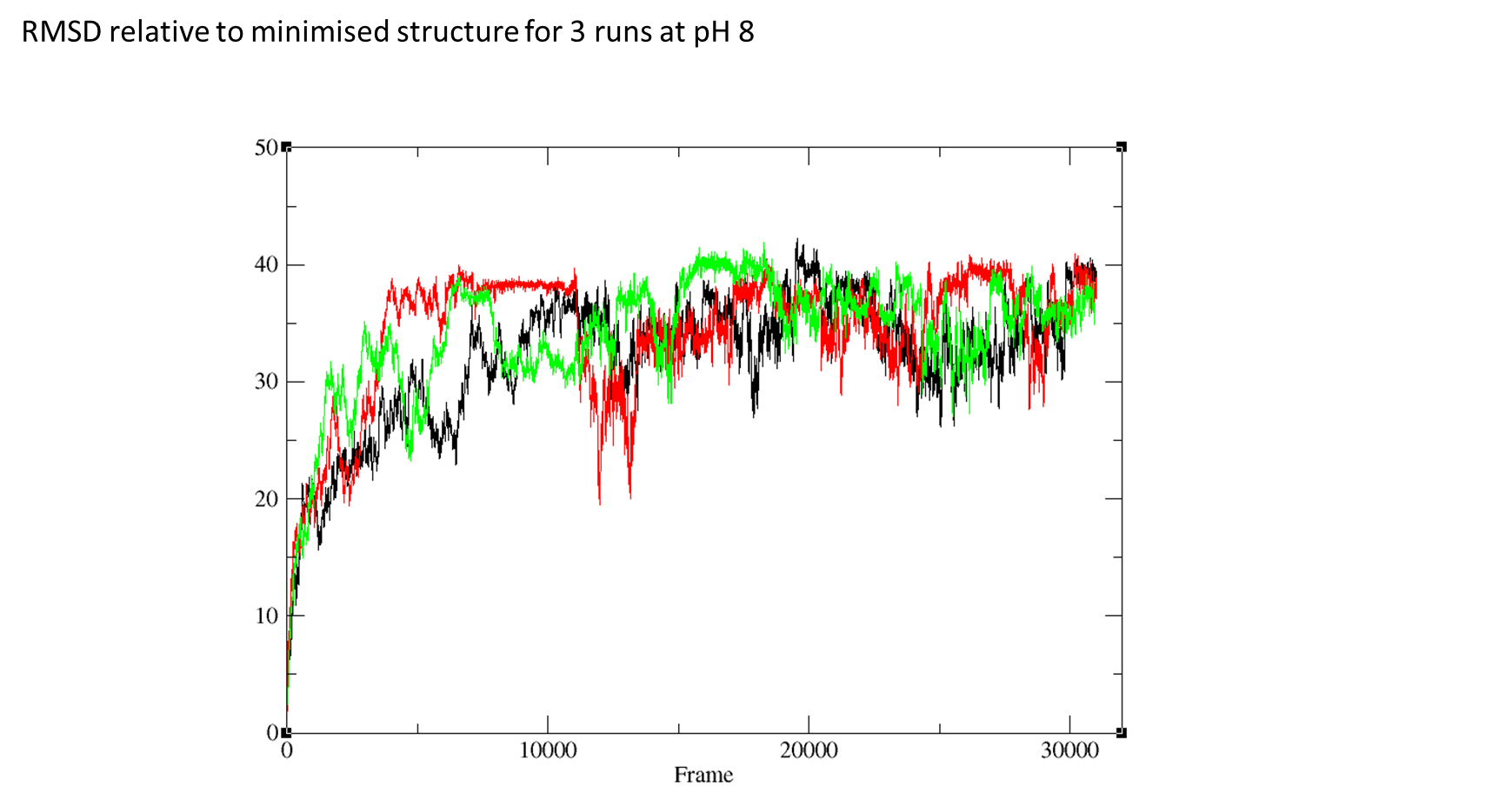


RMSD / Angstrom

**Figure S5** Snapshots of different Rg regimes of Aβ1-40 at pH 6. Histidine residues are shown as lines.

| 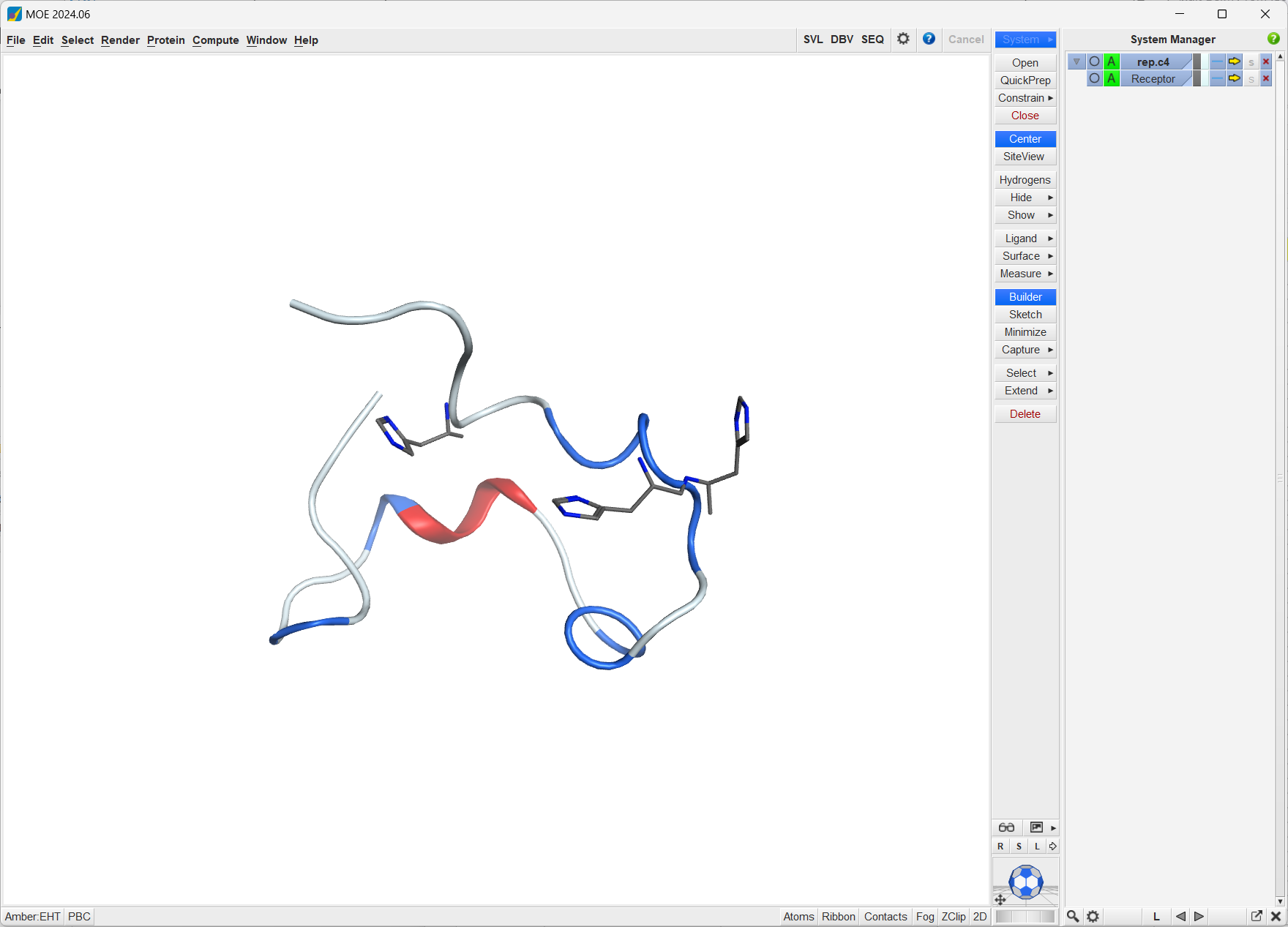 |
| --- |
| Rg = 10.1 Å |
| 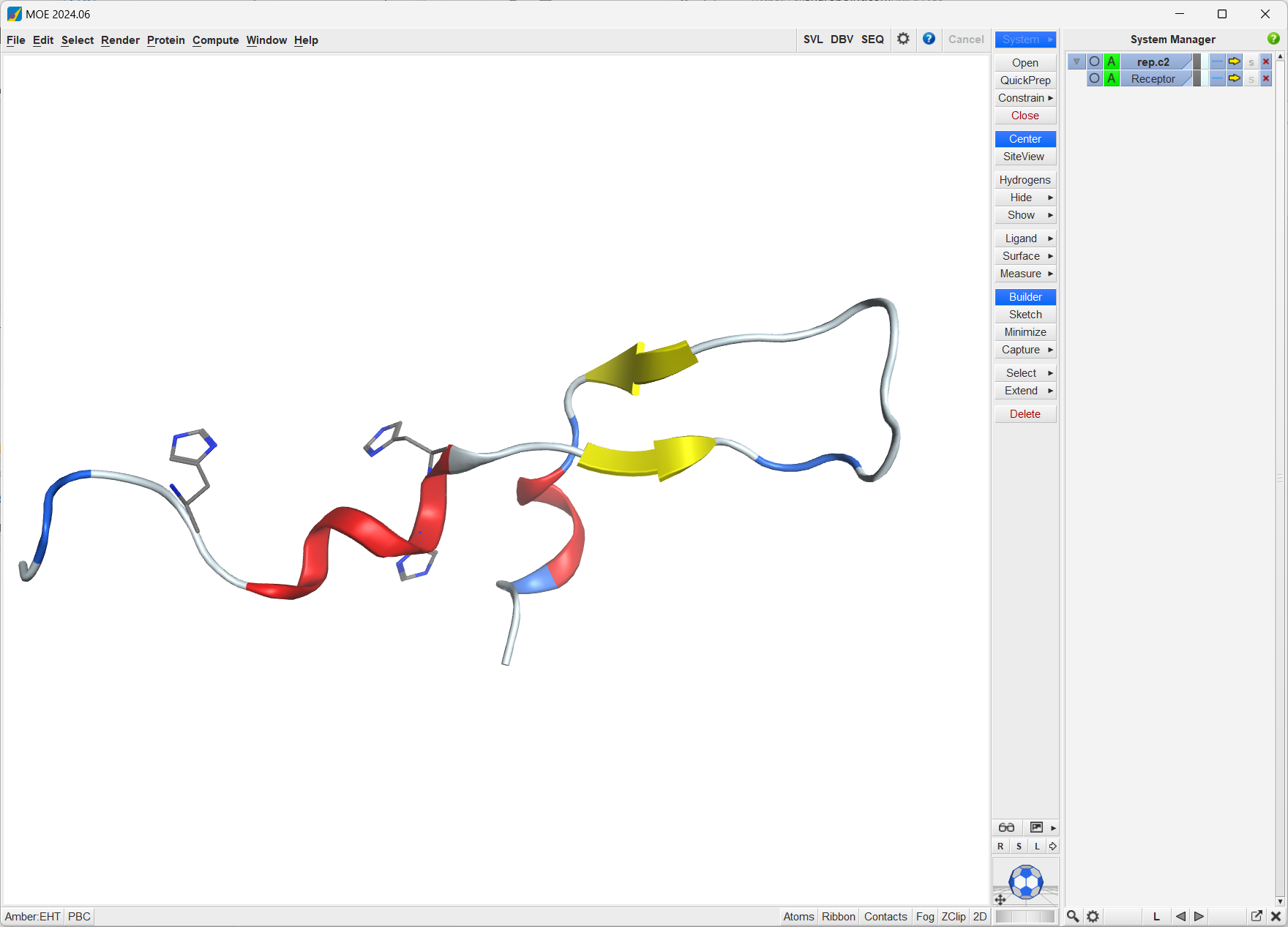 |
| Rg = 13.4 Å |
| 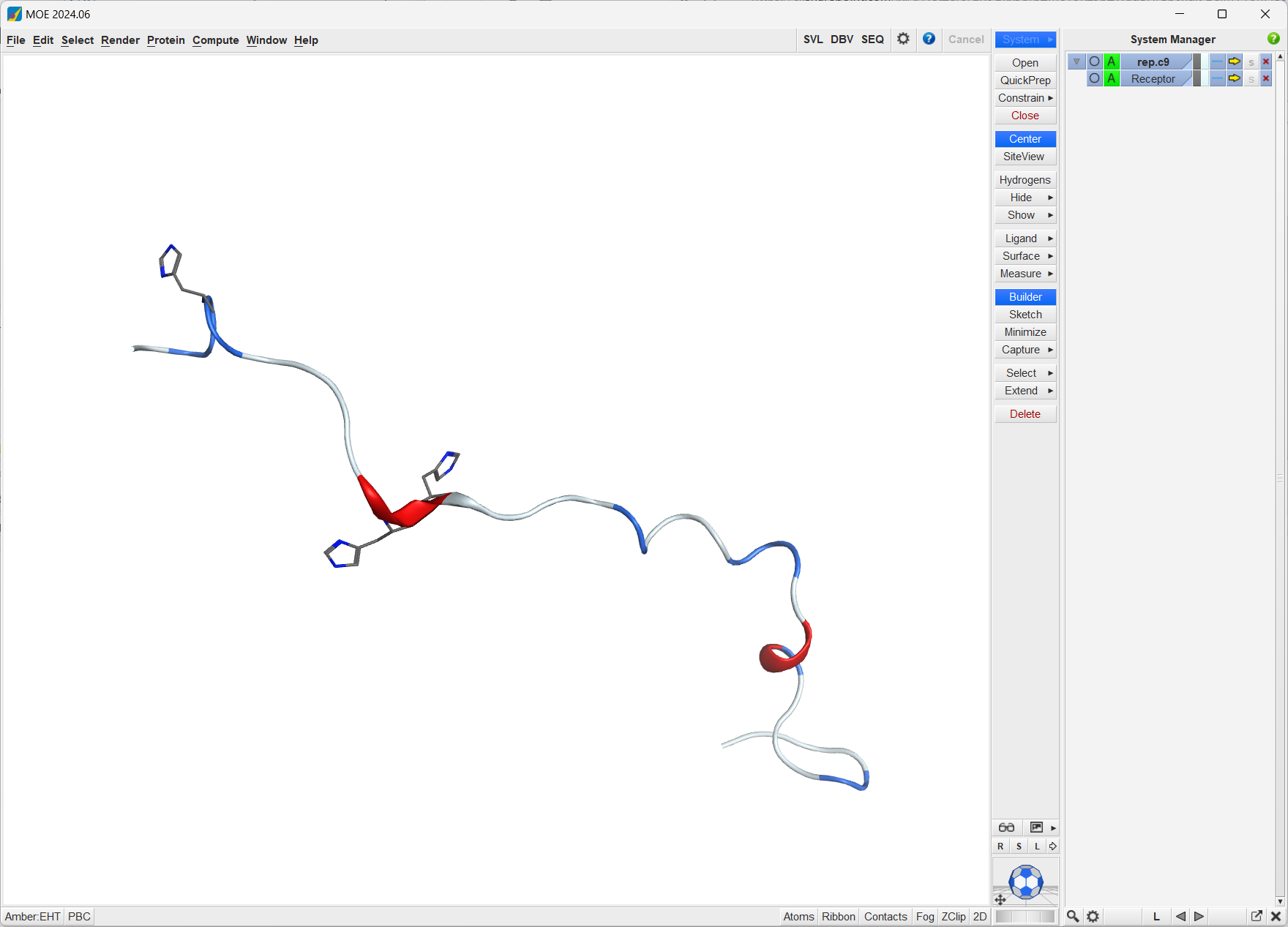 |
| Rg = 20.7 Å |

**Figure S6** Salt-bridge occurrences for His at pH=7 and 8


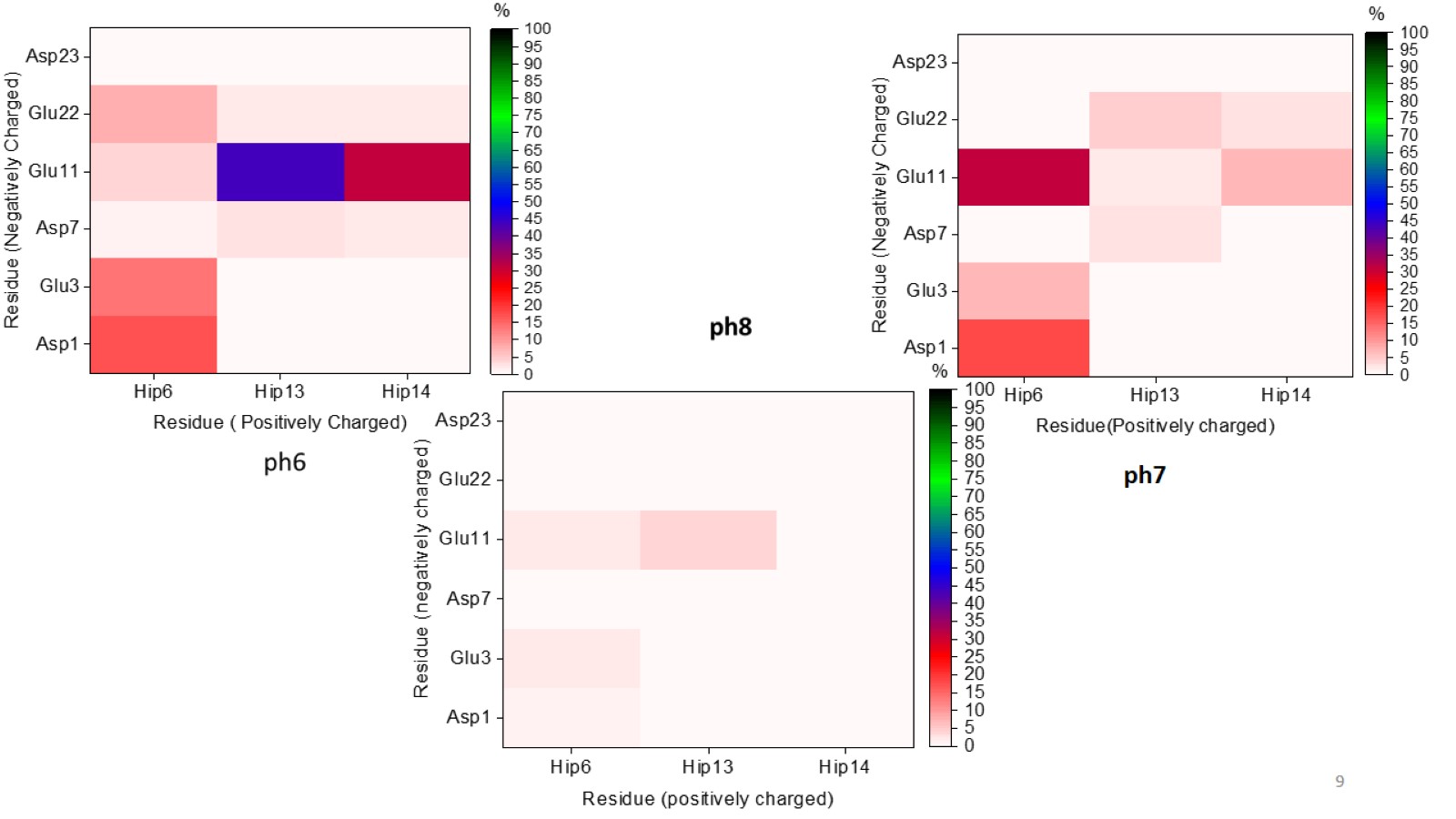


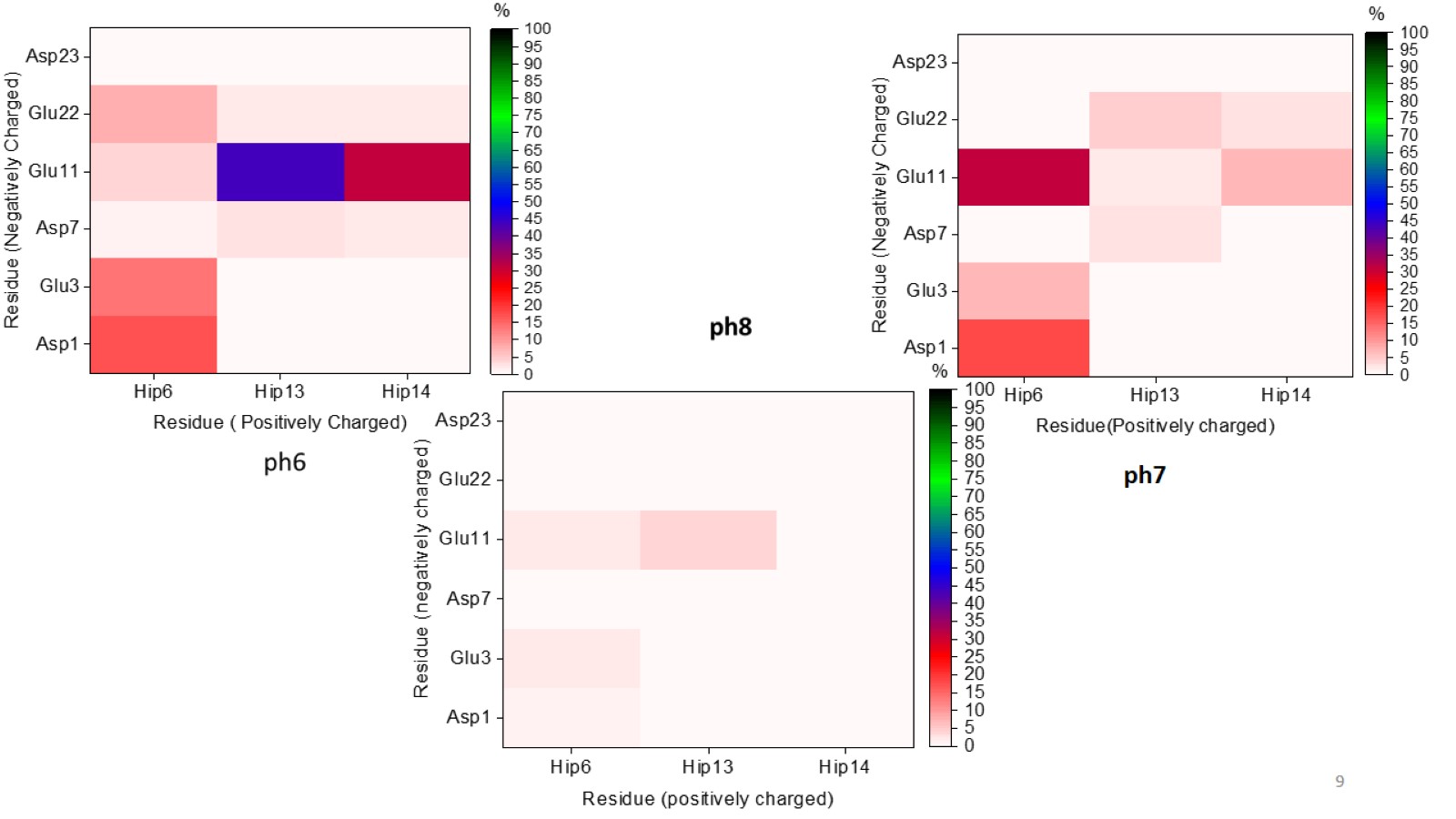


**Figure S7** Root mean square fluctuation for Ab40 at pH = 6, 7, 8
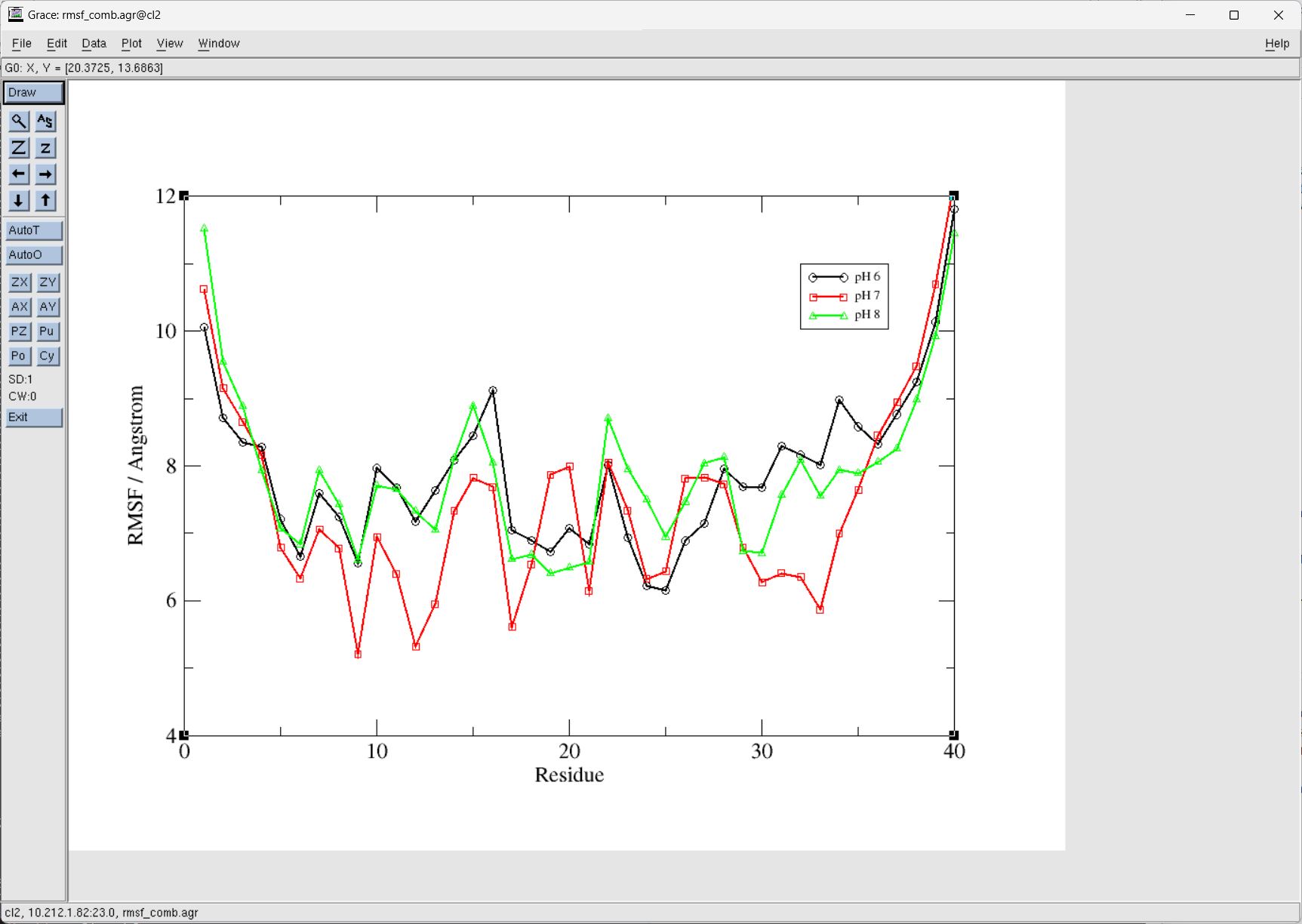


**Figure S8** Remaining nine clusters for each pH; Red: helix, Blue: turn, Yellow: strand


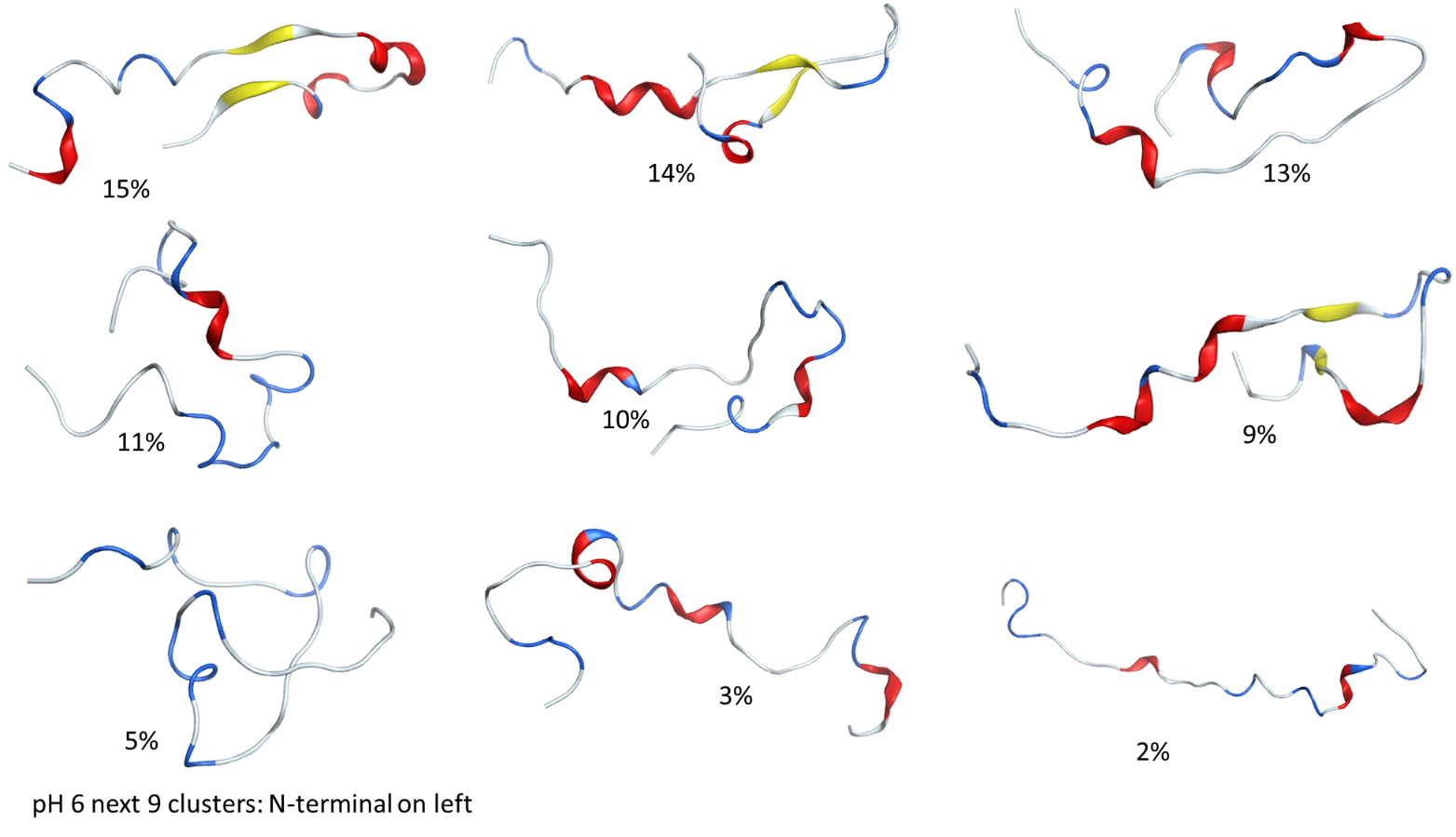


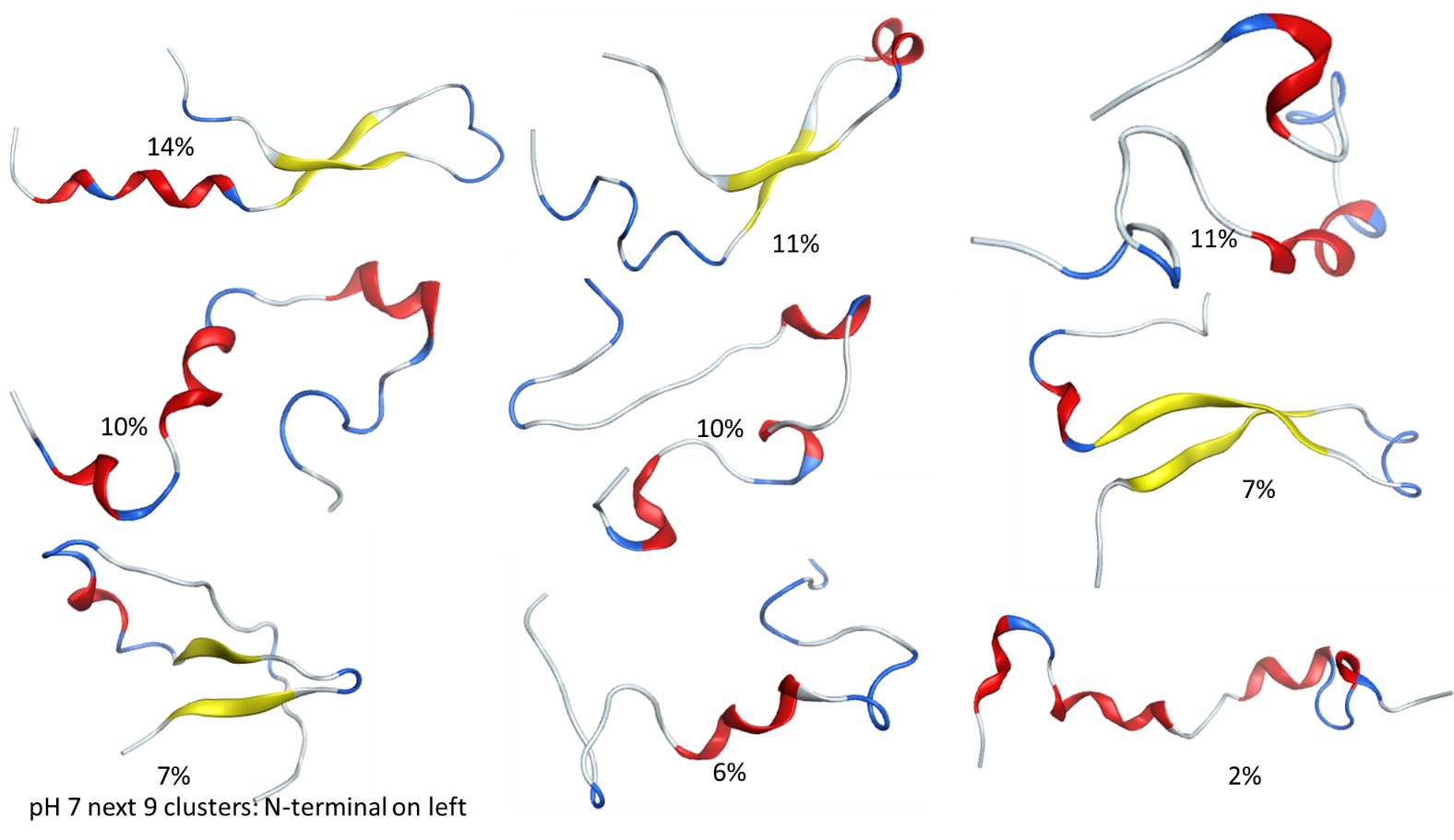


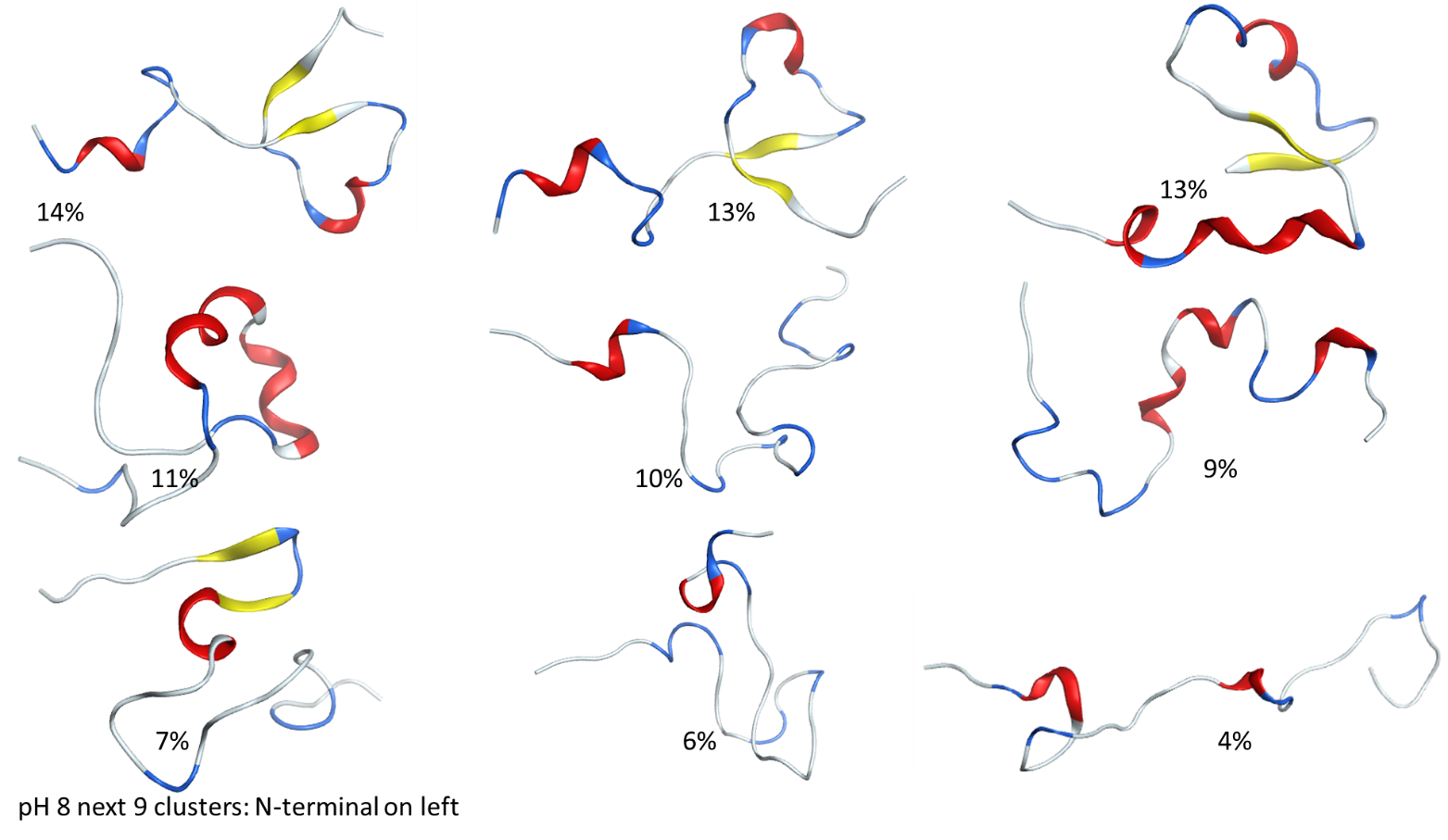


**Figure S9** Potential ionic interactions of His in most populated cluster at pH 6


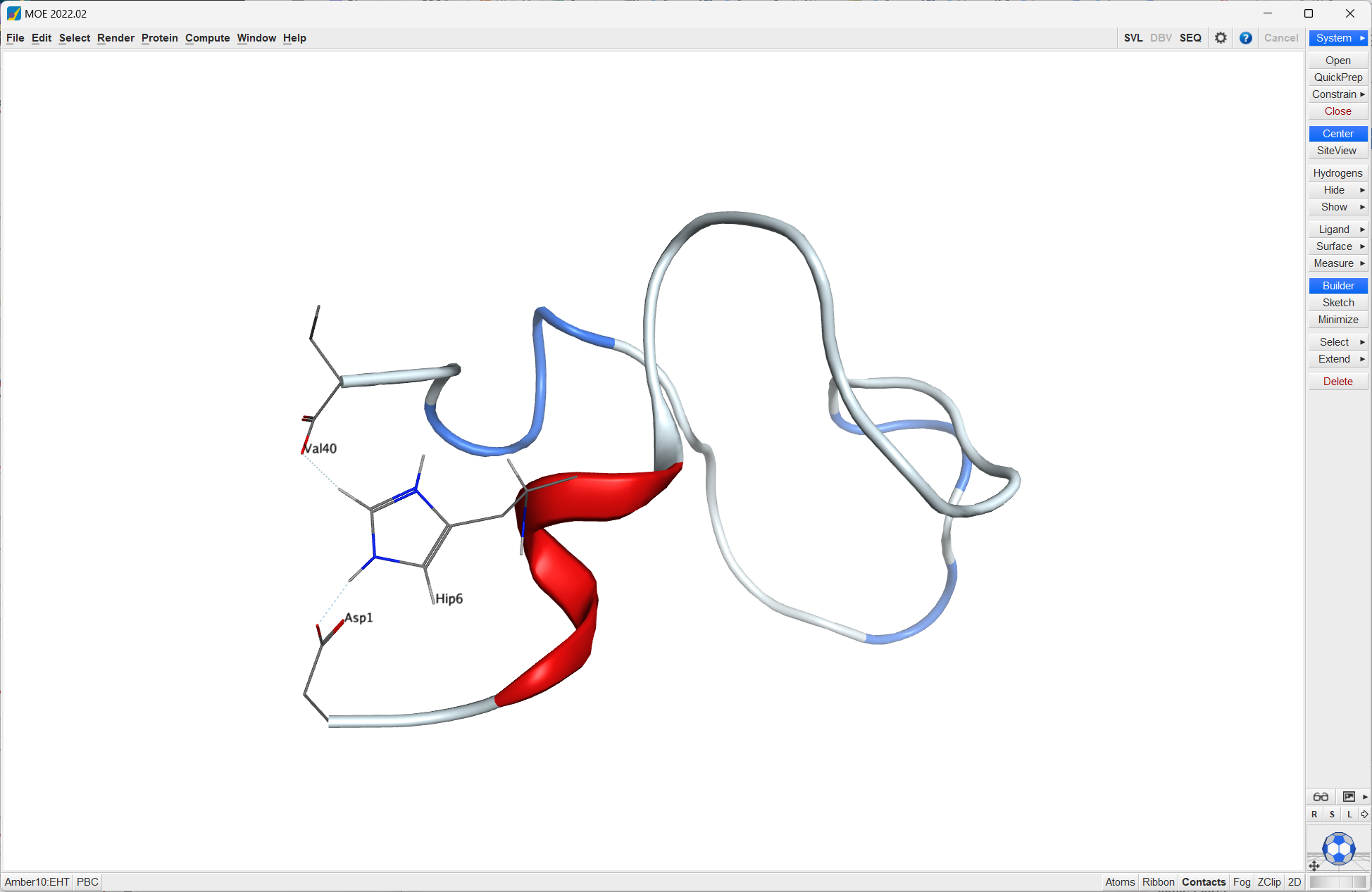


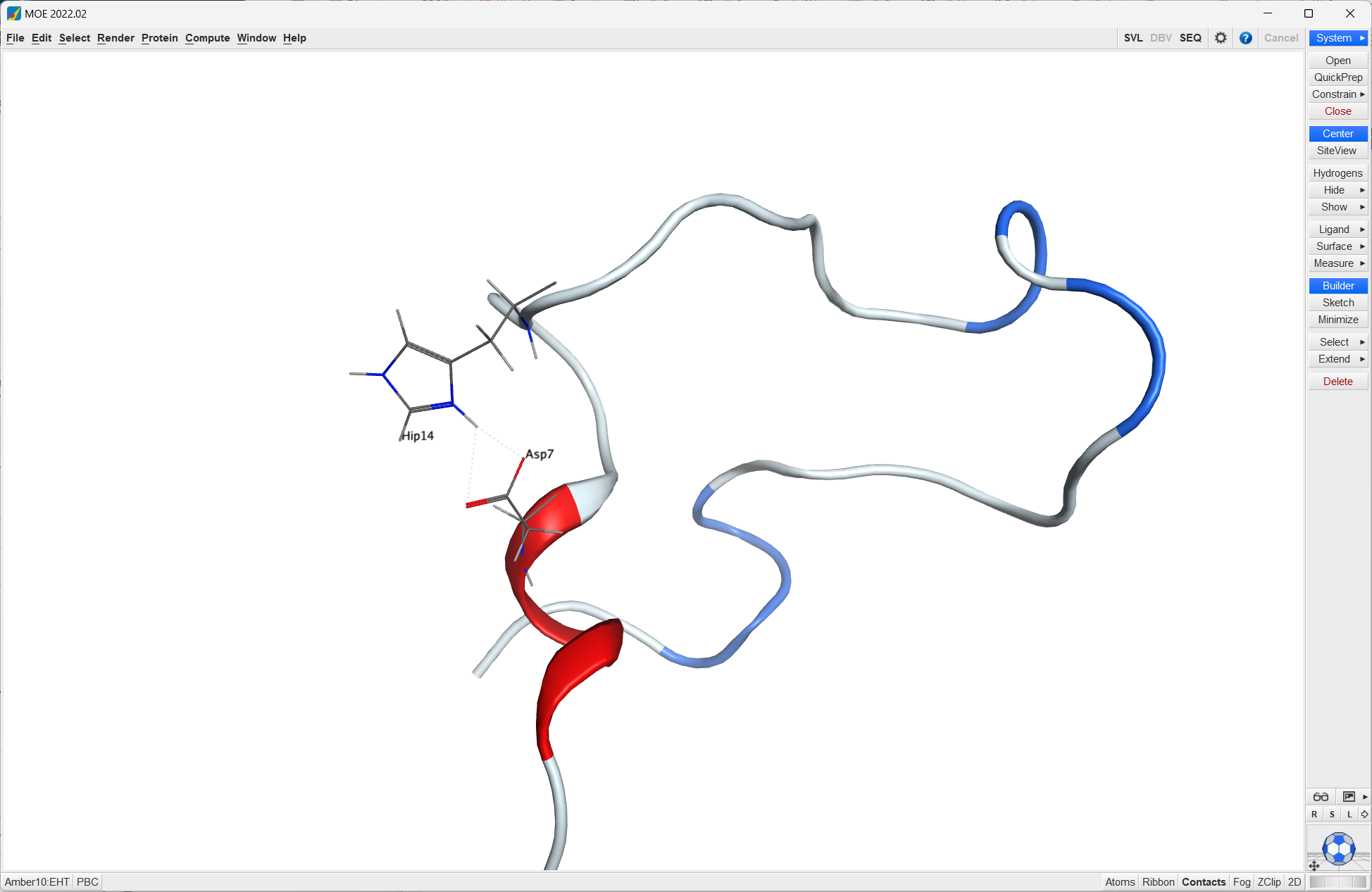


**Figure S10** Convergence of Rg for Cu component I over 1000 ns. Points are mean over 100 ns window; Error bars show standard deviation of Rg within each window.


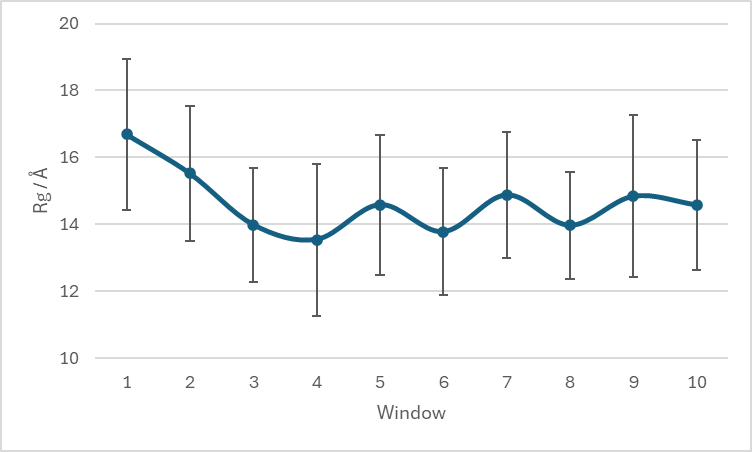


**Figure S11** Remaining nine clusters for each Zn and Cu, type I and II; Red: helix, Blue: turn, Yellow: strand


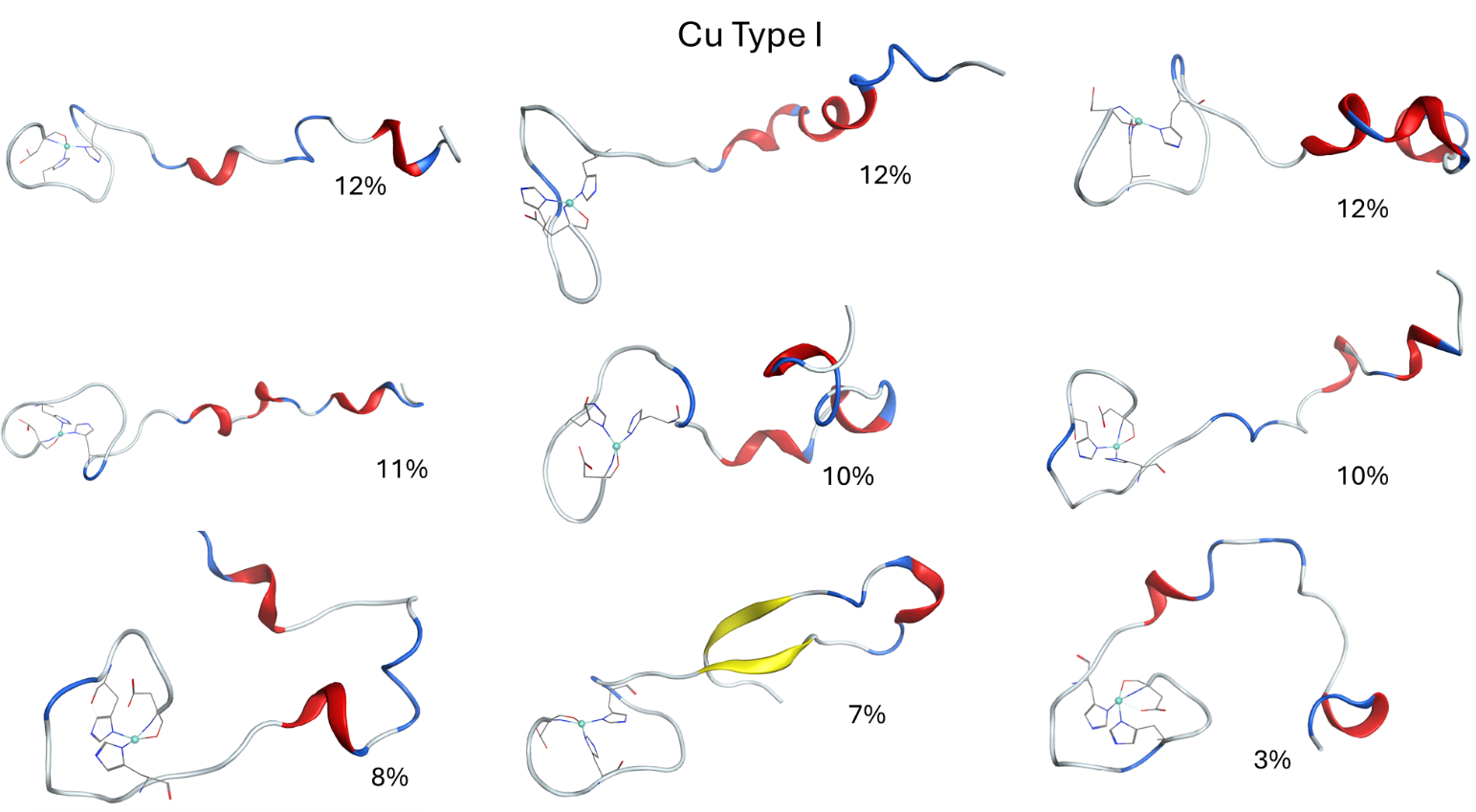


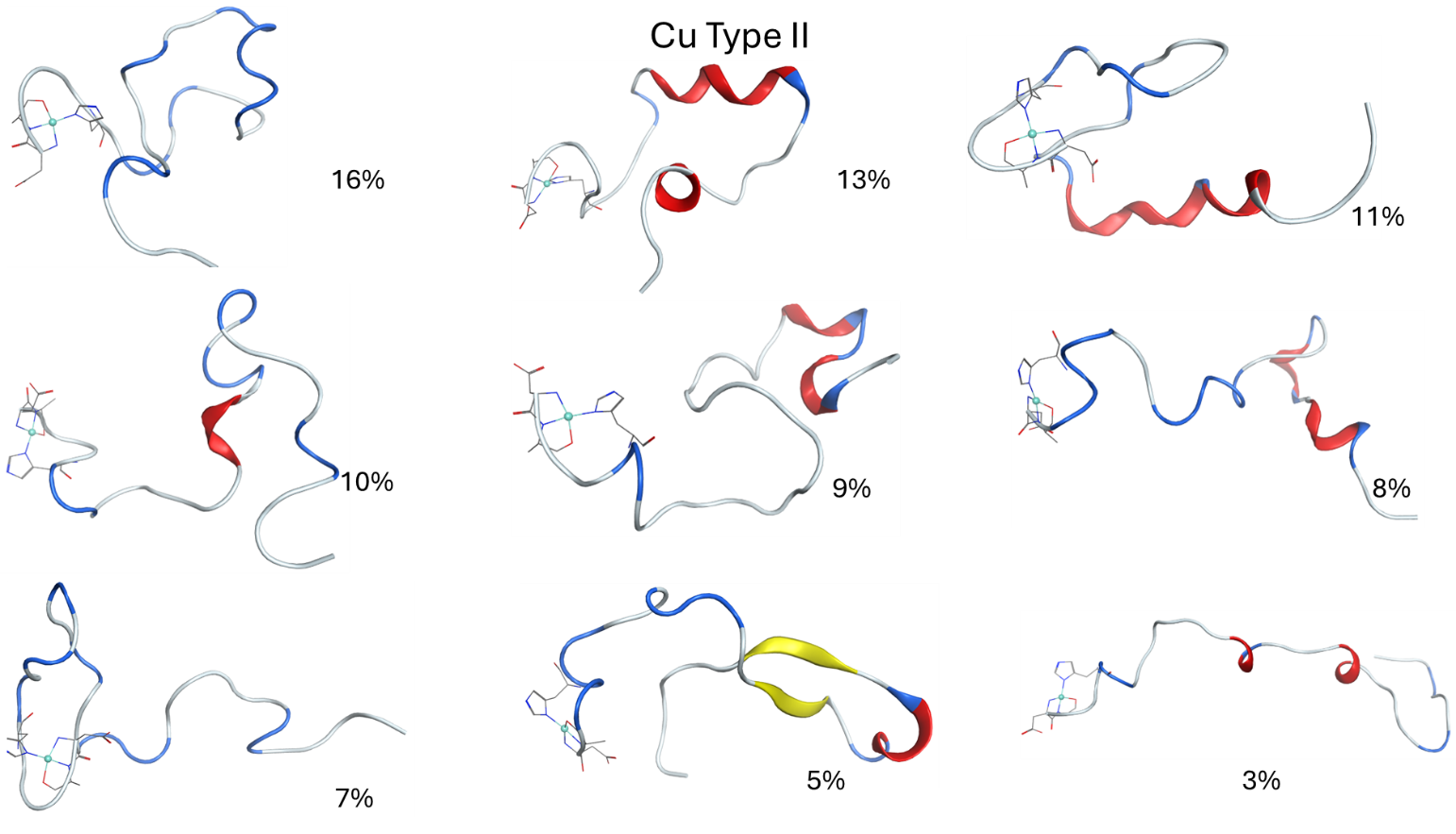


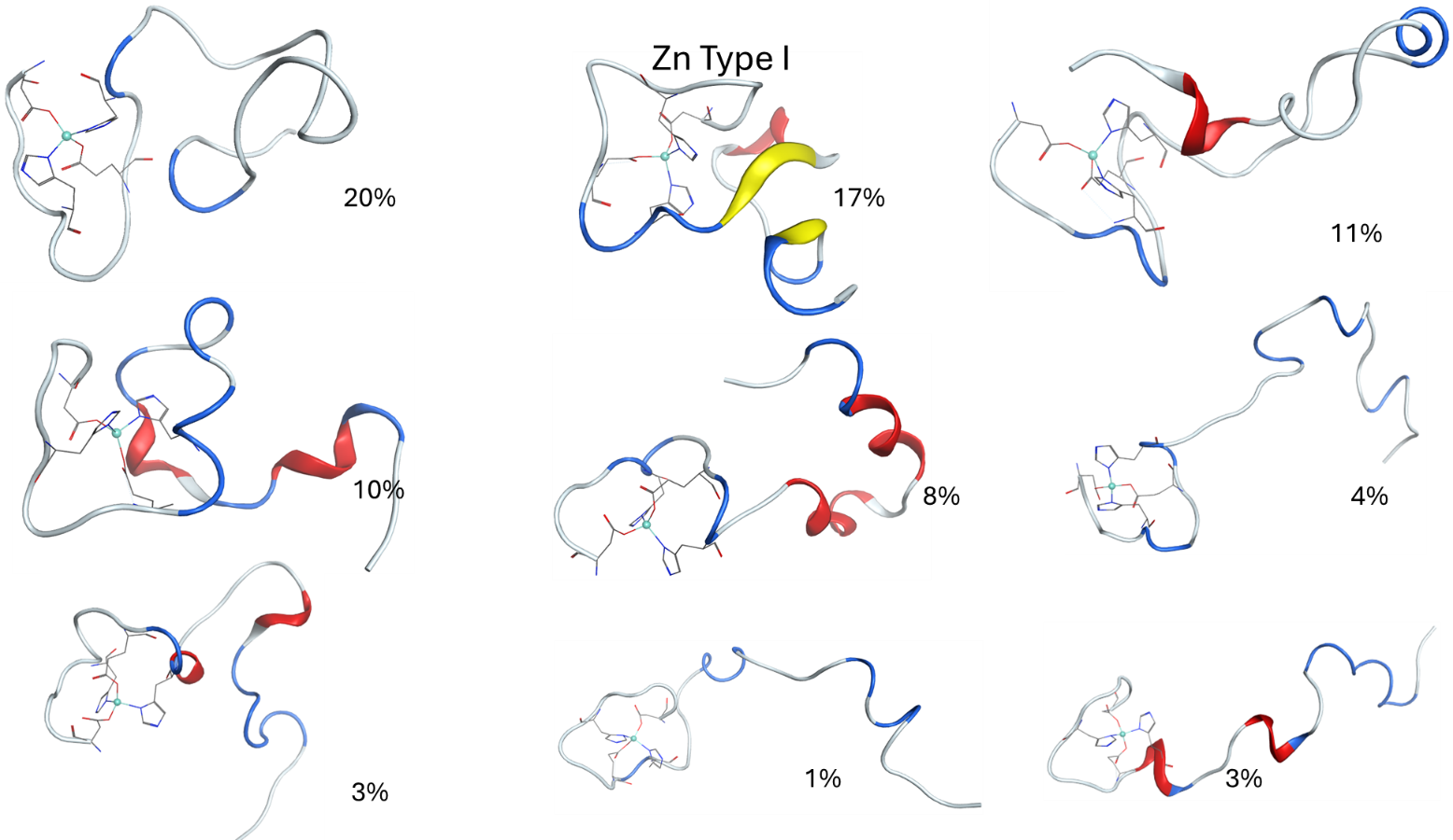


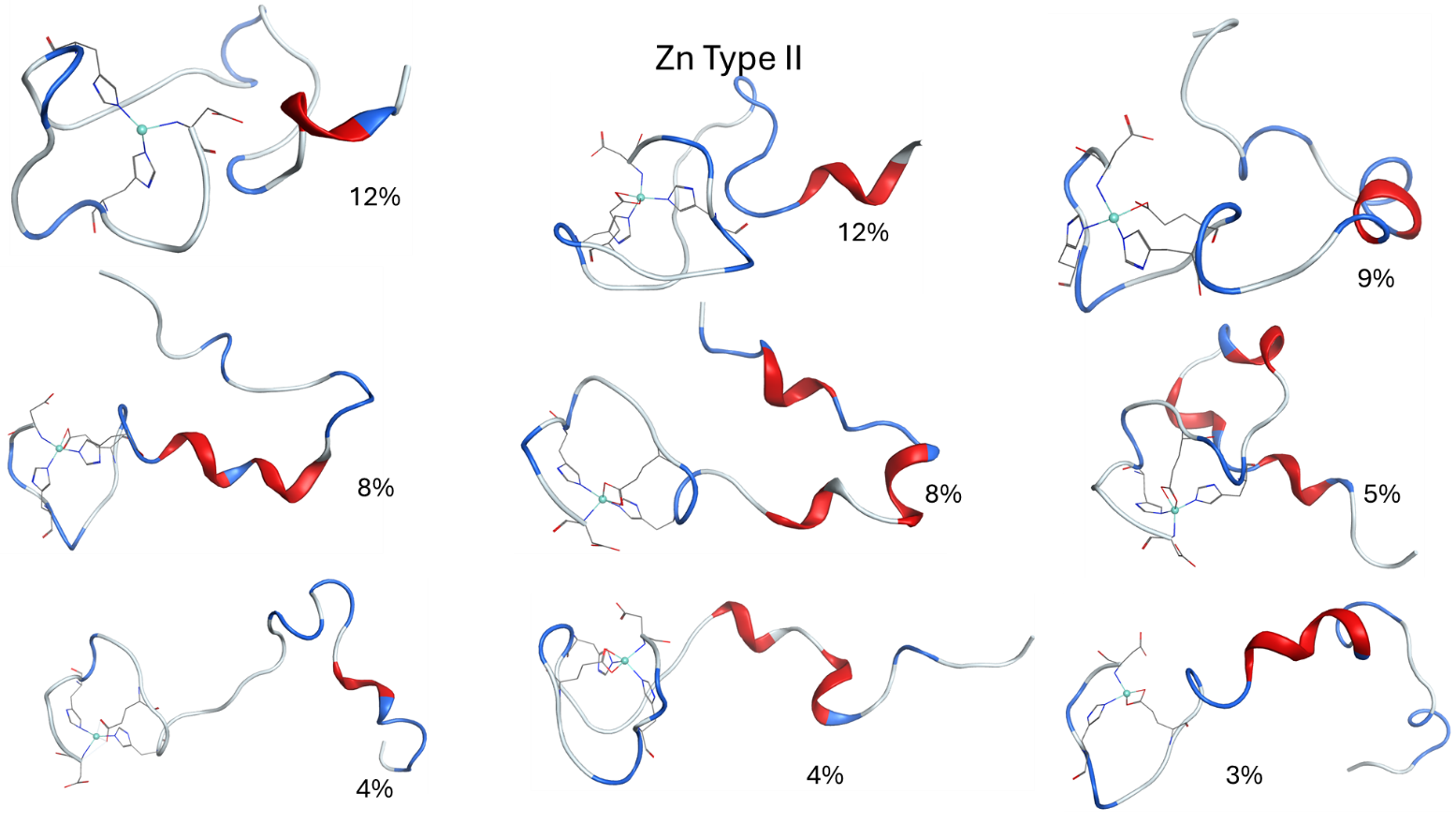

Supplement: Supplementary file 1 — Supporting Information [file CHEM-31-e202500547-s001.docx]
